# Clinical progression parameters associated with SARS-CoV-2, influenza, and respiratory syncytial virus infections in a large US integrated healthcare population

**DOI:** 10.1371/journal.pcbi.1013723

**Published:** 2025-11-19

**Authors:** Noah T. Parker, Vennis Hong, Gregg S. Davis, Magdalena Pomichowski, Iris A. Reyes, Fagen Xie, Nicola F. Mueller, Isabel Rodriguez-Barraquer, Sara Y. Tartof, Joseph A. Lewnard

**Affiliations:** 1 Department of Epidemiology, Johns Hopkins University Bloomberg School of Public Health, Baltimore, Maryland, United States of America; 2 School of Public Health, University of California, Berkeley, Berkeley, California, United States of America; 3 Department of Research and Evaluation, Kaiser Permanente Southern California, Pasadena, California, United States of America; 4 Division of HIV, Infectious Diseases, and Global Medicine, University of California, San Francisco, San Francisco, California, United States of America; 5 Chan Zuckerberg Biohub, San Francisco, California, United States of America; 6 Department of Health Systems Science, Kaiser Permanente Bernard J. Tyson School of Medicine, Pasadena, California, United States of America; 7 Center for Computational Biology, College of Data Science and Society, University of California, Berkeley, Berkeley, California, United States of America; Northeastern University, UNITED STATES OF AMERICA

## Abstract

Mathematical and computational models are often used to forecast respiratory infectious disease burden, including to inform healthcare capacity. We aimed to characterize pathways of clinical progression associated with SARS-CoV-2, influenza, and respiratory syncytial virus (RSV) infections using data from patients aged 0 to >90 years in an integrated healthcare system, whose encounters were monitored across all levels of acuity spanning virtual, ambulatory, and inpatient care settings. Using parametric survival models, we estimated probabilities of progression and distributions of time to progression from each setting to all higher-acuity settings on a cascade encompassing the following classes of events or encounters: symptoms onset; diagnostic testing; telehealth or other virtual care appointment; outpatient physician office visit; urgent care presentation; emergency department presentation; hospital admission; mechanical ventilation; and death. Our analyses included data from 59,668, 22,705, and 1,668 episodes associated with positive SARS-CoV-2, influenza, and RSV tests, respectively, between 1 April 2023 and 31 March 2024. First clinical encounters occurred in inpatient settings for only 4.7%, 3.4%, and 18.7% of SARS-CoV-2, influenza, and RSV episodes, respectively, with median times (interquartile range) of 6.8 (3.6-13.2), 6.6 (3.5-12.1), and 6.4 (3.8-10.6) days from symptoms onset to admission. Overall, 7.9% of SARS-CoV-2 episodes, 5.8% of influenza episodes, and 33.8% of RSV episodes resulted in inpatient admission, ventilation, or death. Between 40.4-62.1%, 71.6-87.3%, and 47.9-58.7% of SARS-CoV-2, influenza, and RSV infections, respectively, had encounters in lower-acuity virtual care, outpatient, or urgent care settings. For all three viruses, the proportions of cases receiving care at each level of acuity increased with older age and greater numbers of comorbid conditions. Median durations of hospital stay were 4.2 (2.6, 7.3), 4.0 (2.3, 6.8), and 4.3 (2.5, 7.4) days for SARS-CoV-2, influenza, and RSV episodes resulting in admission. These estimates provide a basis for modeling real-world clinical care requirements and the progression of respiratory viral infections.

## Author summary

Models of respiratory infections such as SARS-CoV-2, influenza, and RSV are used to forecast disease burden and plan the allocation of healthcare resources. However, limited data are available addressing patterns of healthcare utilization among patients with these infections. Using electronic healthcare records from an integrated healthcare system, we estimated probabilities and rates of progression from lower-acuity states, such as virtual or outpatient visits, to increasingly higher-acuity states including inpatient admission, ventilation, and death. We quantified associations of demographic and clinical risk factors with progression probabilities for each infection. We provide a databank containing fitted distributions for progression to inform infectious disease modeling.

## Introduction

Acute respiratory illnesses (ARIs) caused by SARS-CoV-2, influenza, and respiratory syncytial virus (RSV) are important contributors to morbidity and mortality in the United States and globally [1–3]. Anticipating healthcare utilization associated with ARIs is an objective of both public health agencies and healthcare delivery organizations. Mathematical and computational models used to forecast ARI burden are often trained using data from either syndromic surveillance or reported cases, hospital admissions, and deaths associated with each infection [4]. Such models employ diverse frameworks, often including mechanistic approaches simulating the natural history and transmission dynamics of infection [5], multiplier approaches anticipating care utilization needs at differing levels of acuity [6], and forecasting or nowcasting approaches based on time series [7–9]. To inform capacity planning—including decisions around the allocation of personnel, space, medications, laboratory infrastructure, and other resources—such models require realistic parameters concerning the likelihood and time course of cases’ healthcare utilization [10].

Despite this need, few real-world data sources address clinical care trajectories associated with ARIs due to SARS-CoV-2, influenza, and RSV. While parameters such as the proportion of SARS-CoV-2 infections resulting in hospital admission or death [11,12] and durations of hospital stay [13,14] were estimated in numerous settings during the early phases of the COVID-19 pandemic, fewer studies have addressed utilization patterns in lower-acuity healthcare settings such as ambulatory clinics and emergency departments, where the greatest numbers of all medically-attended cases receive care. Moreover, updated epidemiological parameter estimates for recent SARS-CoV-2 variants and in populations with widespread immunity are not widely available. These challenges are equally pronounced in efforts to model seasonal influenza and RSV. Although some studies have aimed to characterize reporting pyramids addressing symptomatic or medically attended, hospitalized, and fatal influenza cases for contexts of seasonal [15] and pandemic influenza [6,16], these studies have drawn on data from disparate sources and settings, and do not address time-to-event parameters that are likewise critical to forecasting.

We aimed to characterize pathways of clinical progression during ARIs associated with SARS-CoV-2, influenza, and RSV. We analyzed data from patients enrolled in capitated, managed care plans within an integrated healthcare system in southern California. This rich data source allowed us to monitor patient encounters across all levels of acuity spanning virtual, ambulatory, and inpatient care settings. We quantified how patients progress through the different settings of care using parametric survival models among all ascertained infections. The outputs of this analysis provide a basis for modeling clinical burden and healthcare system impacts of SARS-CoV-2, influenza, and RSV infections.

## Methods

### Ethics statement

This study was reviewed and approved by the Kaiser Permanente Southern California institutional review board, which granted a waiver of informed consent for retrospective analysis of EHR data.

### Overview of the modeling approach

**Fig 1:**
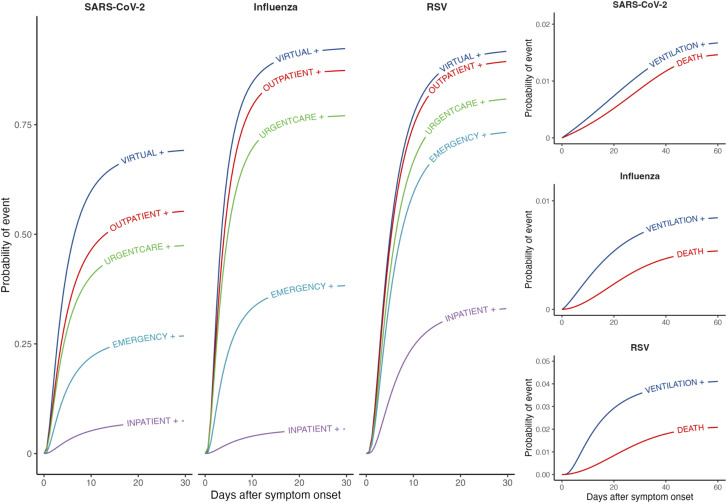
Clinical care cascade for acute respiratory illnesses associated with SARS-CoV-2, influenza, and RSV infections. We illustrate cumulative distribution functions from best-fitting models (defined by Akaike information criterion values) for times from symptom onset to progression to (or beyond) each acuity threshold. Plus signs (+) next to states indicate receiving care at the indicated level or a higher-acuity level of care. Right-hand panels illustrate cumulative distribution functions for rare outcomes (mechanical ventilation and death).

We defined a cascade of clinical progression wherein we aimed to estimate (a) the probability that an individual observed at any state along this cascade would progress to a higher-acuity state, and (b) the distributions of times to progression from lower-acuity to higher-acuity states (**Figs 1**; S1 File). States were characterized as the highest-acuity settings where individuals had received care for ARI at a given point during their infection. We defined the states in order of increasing acuity as: any infection or symptomatic infection without associated healthcare utilization (besides testing); telehealth or other virtual care appointment; outpatient physician office visit; urgent care presentation; emergency department presentation; hospital admission; mechanical ventilation; and death. Our analyses account for the fact that individuals may progress from lower- to higher-acuity states without being intercepted via healthcare encounters at intermediate levels between these origin and destination states. As ongoing receipt of care in lower-acuity settings does not provide a basis for inferring recovery, our analysis considers “forward” transitions to care in higher-acuity settings only.

We fit parametric survival models to jointly estimate the probabilities of progression and the distributions of time to progression from each state to all higher-acuity states on the cascade. We modeled three classes of transitions aiming to inform distinct forecasting applications. First, we modeled an individual’s most proximal progression event from each originating state on the cascade, aiming to characterize typical pathways of care utilization (e.g., the probability and time-to-event for the first hospital admission following emergency department presentation). Secondly, we modeled an individual’s total probability of progression to each state or more severe states from all lower-acuity originating states on the cascade, aiming to inform projections of total demand at each level of acuity within a patient cohort observed at any point in time (e.g., the probability and time-to-event for care receipt at an urgent care facility following a virtual care appointment). Lastly, we aimed to estimate durations of hospital stay following inpatient admission. We explored the association of progression risk and progression rates with individual epidemiologic and demographic characteristics by including covariates in survival models.

We share parameters of all fitted models for re-use within the supplementary materials and via an online repository (https://github.com/ntparker3/Resp_params).

### Setting

We used data from healthcare encounters among members of Kaiser Permanente Southern California (KPSC), an integrated healthcare system providing care across virtual, outpatient, and inpatient settings to roughly 4.7 million individuals throughout southern California during the study period. Members of KPSC enroll through a combination of employer-sponsored, pre-paid, and government-subsidized insurance plans, and broadly reflect the socioeconomic and racial and ethnic diversity of the area’s population [17,18]. Electronic health records (EHRs) capture clinical notes, diagnoses, laboratory results, and prescriptions for care received at KPSC facilities, while insurance claims capture out-of-network care, enabling near-complete ascertainment of healthcare delivery for members. Testing for the studied viral pathogens is conducted by in-house clinical laboratories, with results linked to patient EHR data via unique patient identification numbers. Medically-supervised deaths are tracked via EHR, while medically-unsupervised deaths are reconciled with health plan administrative and clinical databases, member proxy reporting, Social Security Administration vital status data, and California death certificates.

### Study population and episode definition

We conducted event-level analyses of ARIs associated with SARS-CoV-2, influenza, and RSV among individuals of any age who tested positive for each pathogen by molecular or antigen-based assays in any clinical setting between 1 April 2023 and 31 March 2024. We selected this study period to ensure results were not impacted by disruptions in routine care delivery associated with earlier emergency phases of the COVID-19 pandemic. Additionally, screening for SARS-CoV-2 infection at the point of hospital admission or other healthcare encounters was no longer undertaken by KPSC during the study period. While co-infections were exceptionally rare, we allowed overlapping episode periods to contribute to observations for each unique virus identified. We limited the study population to individuals with ≥1 year of continuous enrollment in a Kaiser health plan before their index test (allowing for enrollment gaps up to 45 days in length, as captured from membership enrollment and disenrollment dates) to ensure accurate characterization of individuals’ baseline health status from prior-year utilization. For children aged <1 year, this requirement applied to parents.

We defined index tests for each ARI episode as the date of specimen collection associated with the first positive test for each pathogen during the study period. Symptoms data (presence of and onset dates for fever, cough, headache, fatigue, dyspnea, chills, sore throat, myalgia, anosmia, diarrhea, vomiting or nausea, and abdominal pain within 14 days before testing) were solicited at the point of testing for all individuals who received SARS-CoV-2 tests during the study period. Symptoms were recorded for most ARI episodes, as nearly all individuals who received influenza or RSV tests within KPSC were previously or concurrently tested for SARS-CoV-2. We supplemented structured data from symptoms questionnaires with searches of free-text EHR fields via a previously described natural language processing (NLP) algorithm [19] to characterize the presence and onset times of symptoms using all available information. We defined the date of symptoms onset as the earliest recorded date within 14 days before to 30 days after testing for each episode.

We characterized ARI episodes using data from all healthcare encounters occurring between the date of symptoms onset (or up to seven days before the date of testing, if symptoms were not recorded at the time of testing) and 30 days after the testing date. We defined dates of progression to each state as the first date at which individuals received care in the associated clinical setting, including at least one ARI-related diagnosis code (S1 Table in S1 File). We excluded any care utilization events where ARI codes were not assigned to ensure that healthcare encounters unrelated to an ongoing ARI episode were not interpreted as indicators of disease progression.

### Statistical analysis

We fit parametric survival models using the *flexsurv* package [20] in R (version 4.4.2; R Foundation for Statistical Computing, Vienna, Austria). We estimated parameters corresponding to assumptions that times-to-event followed exponential, Weibull, Gompertz, Gamma, generalized Gamma, and log normal distributions for all transitions. The modeled distributions invoke differing assumptions about underlying rate or processes of progression across clinical care settings. The exponential distribution assumes events occur independently with a constant rate, an assumption that the Weibull and Gompertz distributions relax by allowing rates to vary over time. The Gamma distribution defines times to progression as the sum of exponentially-distributed event times, which in our application may correspond to underlying disease progression events prompting receipt of higher-acuity care, while the generalized Gamma combines the refinements of both the Weibull and Gamma distributions. The log normal distribution, in contrast, lacks a similar mechanistic interpretation but may provide a good approximation to observed event time distributions. For brevity, we describe results for models with the lowest Akaike information criterion (AIC) values for each transition in this manuscript, and present parameter estimates for all distributions evaluated within the accompanying code base (https://github.com/ntparker3/Resp_params). Practitioners, however, should consider the mechanistic assumptions underlying differing time-to-event distributions alongside or in lieu of model selection criteria when choosing among the differing parameterizations generated.

For models of the most proximal transition from each state, we followed for progression events within 20 days after dates of entry into each originating state. We considered observations to be censored if no progression event occurred within 20 days. As a sensitivity analysis, we also present estimates for models including follow-up through 60 days for these transitions. We used mixture models to estimate probabilities of and times to the most proximal progression event from each originating state. These models defined distinct rates for progression between each originating state and all higher-acuity states, and handled progression to each state as a competing risk. This framework corresponded to the interpretation that progression to a higher-acuity state of illness could precede progression to intermediate states along the same cascade.

For models of individuals’ total risk of progression to each acuity state (or higher-acuity states), we followed for progression events within 60 days after dates of entry into each originating state. When individuals experienced care corresponding to multiple states on the same day (e.g., an emergency presentation leading to hospital admission), we defined the highest-acuity state observed as the outcome. In contrast to analyses for individuals’ most proximal transition, models for individuals’ risk of progression to each state—cumulatively across all intermediate pathways of care—did not require a competing-risks framework. For these analyses, we instead recorded progression as occurring when individuals experienced the outcome of interest or one signifying receipt of higher-acuity care.

To estimate hospital lengths of stay, we fit parametric survival models defining admission dates as originating events and dates of discharge with any disposition or in-hospital mortality as the outcome; we modeled consecutive admissions with same-day readmissions as continuous hospitalization events. We also used mixture models defining competing risks for death and discharge to separately estimate durations of hospitalization according to individuals’ clinical outcome.

Differences in rates across patient subgroups could impact the validity of population-wide estimates. We therefore repeated the analyses described above to estimate subgroup-specific risks and rates of progression based on age, sex, race/ethnicity, vaccination status, Charlson comorbidity index (a weighted index of 19 different comorbidities where a higher score indicates a greater risk of mortality [21]; S2 Table in S1 File), and community-level socioeconomic status, as measured by census tract-level neighborhood deprivation index values derived from the 2017–2021 5-year estimates of the American Community Survey [22,23]. We categorized continuous covariates according to the distributions presented in **Table 1**. We fit parametric survival models allowing variation across covariate strata in both the probability of progression and the location parameter for times-to-event for each modeled distribution. As for our primary analyses, we describe results for models yielding the lowest AIC value for each transition in this manuscript, and present parameter estimates for all distributions in the accompanying repository. We evaluated 95% confidence intervals around estimated probabilities of progression and median times-to-events to assess whether differences across groups were statistically or epidemiologically meaningful.

**Table 1:** Individual characteristics by infecting virus.

Characteristic	Cases, by infecting virus, *n* (%)		
	*SARS-CoV-2*	*Influenza*	*RSV*
	*N = 59,670*	*N = 23,375*	*N = 1,668*
Age, years			
0-17	4,489 (7.5)	7,348 (31.4)	920 (55.2)
18-49	23,033 (38.6)	8,475 (36.3)	71 (4.3)
50-59	9,307 (15.6)	2,708 (11.6)	87 (5.2)
60-69	8,930 (15.0)	2,283 (9.8)	146 (8.8)
70-79	8,089 (13.6)	1,708 (7.3)	176 (10.6)
80-89	4,639 (7.8)	701 (3.0)	193 (11.6)
≥90	1,183 (2.0)	152 (0.7)	75 (4.5)
Sex			
Male	24,195 (40.5)	10,541 (45.1)	775 (46.5)
Female	35,475 (59.5)	12,834 (54.9)	893 (53.5)
Race/ethnicity			
White, non-Hispanic	14,557 (24.4)	5,237 (22.4)	482 (28.9)
Asian, non-Hispanic	7,033 (11.8)	2,351 (10.1)	201 (12.1)
Black, non-Hispanic	6,767 (11.3)	2,177 (9.3)	166 (10.0)
Hispanic (any race)	28,114 (47.1)	12,228 (52.3)	764 (45.8)
Pacific Islander	484 (0.8)	165 (0.7)	– –
Native American/Alaska native	115 (0.2)	33 (0.1)	3 (0.2)
Other	859 (1.4)	409 (1.7)	14 (0.8)
Multiple	214 (0.4)	126 (0.5)	8 (0.5)
Unknown	1,527 (2.6)	649 (2.8)	17 (1.0)
Insurance source			
Commercial	30,861 (51.7)	13,073 (55.9)	688 (41.2)
Medicaid	6,201 (10.4)	3,258 (13.9)	299 (17.9)
Medicare	13,358 (22.4)	2,574 (11.0)	400 (24.0)
Pre-paid plans	1,444 (2.4)	718 (3.1)	33 (2.0)
Other	653 (1.1)	190 (0.8)	14 (0.8)
Unknown	7,153 (12.0)	3,562 (15.2)	234 (14.0)
Vaccinations			
0 COVID-19 vaccie doses	6,944 (11.6)	6,751 (28.8)	788 (47.2)
1-2 COVID-19 vaccine doses	11,272 (18.9)	5,459 (23.4)	156 (9.4)
3 + COVID-19 vaccine doses	41,454 (69.5)	11,165 (47.8)	724 (43.4)
Seasonal influenza vaccine received	40,565 (68.0)	12,269 (52.5)	1,287 (77.2)
RSV vaccine received	443 (0.7)	225 (1.0)	27 (1.6)
Neighborhood deprivation index			
NDI < –1	2,565 (4.3)	1,027 (4.5)	78 (4.7)
–1 ≤ NDI < 0	19,632 (32.9)	7,247 (31.0)	535 (32.1)
0 ≤ NDI < 1	20,701 (34.7)	8,112 (34.7)	576 (34.5)
NDI > 1	11,097 (18.6)	4,637 (19.8)	279 (16.7)
Unknown	5,675 (9.5)	2,352 (10.1)	200 (12.0)
Charlson comorbidity index			
0	32,336 (54.2)	15,324 (65.6)	834 (50.0)
1-2	16,321 (27.4)	5,748 (24.6)	379 (22.7)
3-5	7,180 (12.0)	1,513 (6.5)	253 (15.2)
6+	3,833 (6.4)	790 (3.3)	202 (12.1)

We enumerate the same characteristics of infections that resulted in an ARI diagnosis in any setting, and infections that reached the threshold of inpatient admission or higher acuity, in S3 Table in S1 File.

## Results

### Descriptive characteristics

Our analyses included data from 348,958 unique KPSC members who received tests for SARS-CoV-2, influenza, or RSV between 1 April, 2023 and 31 March, 2024, among whom we identified 59,670 episodes associated with positive SARS-CoV-2 test results, 23,375 episodes associated with positive influenza test results, and 1,668 episodes associated with positive RSV test results (**Table 1**). Among these episodes, 602 were associated with coinfections (579 SARS-CoV-2 and influenza coinfections, 11 SARS-CoV-2 and RSV coinfections, and 12 influenza and RSV coinfections). In total, 2,737 ARI episodes occurred without associated testing for SARS-CoV-2, influenza, or RSV over the study period, and were not eligible for inclusion in our analyses (S2 Fig in S1 File). The greatest numbers of SARS-CoV-2 and influenza infections occurred among individuals aged 18–49 years (*n* = 23,033 [38.6%] and *n* = 8,475 [36.3%], respectively). For influenza and RSV, a considerable number of episodes also occurred among children aged ≤17 years (*n* = 7,348 [31.4%] and 920 [55.2%], respectively), while 10.7-26.7% of infections with each pathogen occurred among individuals aged ≥70 years (*n* = 13,911 with SARS-CoV-2, *n* = 2,561 with influenza, and *n* = 444 with RSV). Most episodes involving each pathogen occurred among Hispanic individuals of any race or White, non-Hispanic individuals without comorbid conditions (S2 Table in S1 File). Across all three pathogens, roughly half (41.2-55.6%) of all infections occurred among individuals enrolled in commercial insurance plans, and a plurality (10.4-17.9%) occurred among individuals enrolled in Medicaid-sponsored plans. Among SARS-CoV-2 infections, 6,944 (11.6%), 11,271 (18.9%), and 41,454 (69.5%) occurred among individuals who had received 0, 1–2, and ≥3 COVID-19 vaccine doses, cumulatively; 12,269 influenza infections (52.5%) occurred among individuals who had received seasonal influenza vaccination, and few RSV infections (*n* = 27; 1.6%) occurred among individuals who were previously vaccinated against RSV. Characteristics receiving ARI diagnoses in any setting differed from those receiving care in inpatient settings (S3 Table in S1 File).

### Care pathways for SARS-CoV-2

**Fig 2:**
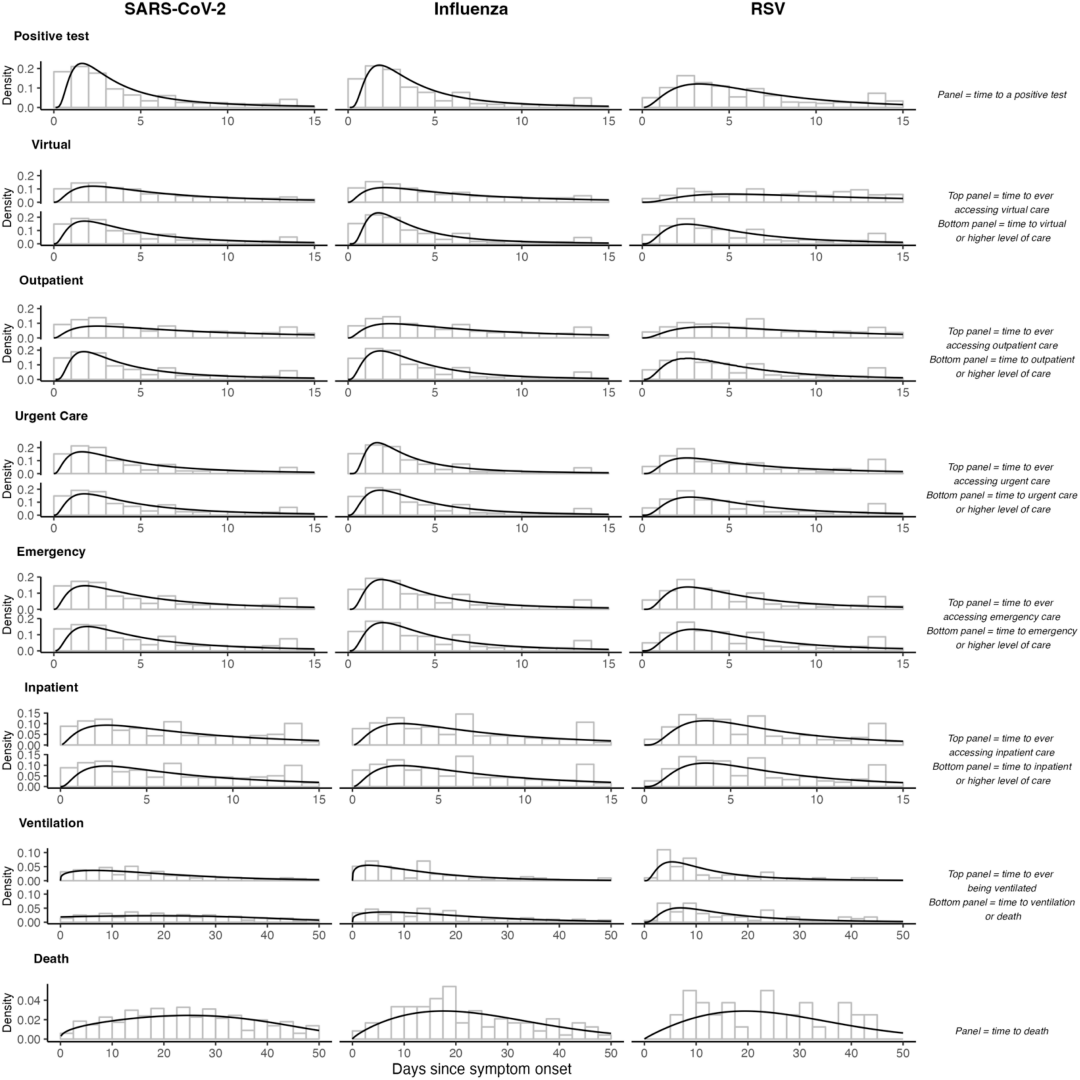
Time-to-event distributions of reaching acuity thresholds from symptom onset for illnesses associated with SARS-CoV-2, influenza, and RSV. For healthcare utilization states with two panels, the top panel illustrates the time from symptom onset to ever seeking care at that specific state, while the bottom panel shows the time from symptom onset to reaching that acuity threshold (seeking care at the state or a state more severe). Panels for *positive test* and *death* show the time from symptom onset to reaching that exact state. Black lines represent the density of the best-fitting distribution, selected by AIC.

For ARIs associated with SARS-CoV-2 infection, the first clinical encounter following symptoms onset most often occurred in urgent care (18.6%) or emergency department (17.9%) settings, followed by virtual care appointments (10.7%), outpatient office visits (7.2%), and inpatient settings (4.7%; **Table 2**; S4 Table; S5 Table in S1 File). Median time from symptoms onset to testing was 3.2 days (**Fig 2**). Among individuals who received virtual care, 21.1% subsequently received care in higher-acuity clinical settings in the following 20 days, with 7.2%, 5.0%, and 7.1%, presenting to outpatient office visits, urgent care facilities, and emergency departments as their next clinical encounter, respectively; 1.9% were admitted to hospital at their next clinical encounter (**Table 2**). For individuals who were admitted at their next clinical encounter after a virtual care appointment, median time to admission was 2.0 days (interquartile range [IQR]: 0.6-5.2). Among individuals who received care at outpatient and urgent care facilities, 4.0% and 1.7%, respectively, were admitted to the hospital at their next clinical encounter after a median of 0.9 and 0.4 days, respectively. We obtained similar estimates in analyses accommodating follow-up through 60 days (S6 Table; S7 Table in S1 File).

**Table 2:** Care utilization pathways associated with each infecting virus using a follow-up period of 20 days.

Originating state	Next outcome	Number of events	Probability of progression, % (95% CI)	Time to progression along indicated transition pathway, days (95% CI)		
				*Median*	*25%ile*	*75%ile*
*SARS-CoV-2 infections*						
Symptoms onset						
	Virtual care	5,796	10.7 (10.5, 10.9)	3.03 (2.96, 3.09)	1.78 (1.74, 1.83)	5.14 (5.03, 5.37)
	Outpatient office visit	3,938	7.2 (7.1, 7.4)	3.64 (3.56, 3.72)	2.13 (2.08, 2.18)	6.21 (6.07, 6.37)
	Urgent care	10,089	18.6 (18.3, 18.9)	3.06 (3.00, 3.12)	1.85 (1.81, 1.89)	5.06 (4.94, 5.18)
	Emergency department	9,723	17.9 (17.6, 18.2)	3.52 (3.44, 3.60)	2.02 (1.97, 2.06)	6.13 (5.99, 6.28)
	Inpatient admission	2,532	4.7 (4.6, 4.8)	5.39 (5.26, 5.51)	2.98 (2.90, 3.05)	8.59 (8.41, 8.78)
	Recovered	22,284	41.0 (40.7, 41.2)	– –	– –	– –
Receipt of test						
	Virtual care	8,206	13.8 (13.6, 13.9)	0.50 (0.49, 0.50)	<0.2	1.52 (1.49, 1.55)
	Outpatient office visit	4,260	7.2 (7.1, 7.2)	<0.2	<0.2	0.53 (0.52, 0.54)
	Urgent care	10,656	17.9 (17.7, 18.1)	<0.2	<0.2	<0.2
	Emergency department	11,690	19.6 (19.4, 19.8)	<0.2	<0.2	<0.2
	Inpatient admission	2,765	4.6 (4.6, 4.7)	<0.2	<0.2	<0.2
	Recovered	21,993	36.9 (36.8, 37.1)	– –	– –	– –
Virtual care						
	Outpatient office visit	1,056	7.2 (7.0, 7.3)	2.83 (2.69, 2.96)	0.76 (0.70, 0.82)	7.40 (7.12, 7.68)
	Urgent care	731	5.0 (4.8, 5.1)	0.92 (0.90, 0.94)	0.26 (0.25, 0.27)	3.28 (3.15, 3.40)
	Emergency department	1,045	7.1 (6.9, 7.3)	0.76 (0.74, 0.77)	0.24 (0.23, 0.24)	2.43 (2.34, 2.52)
	Inpatient admission	285	1.9 (1.9, 2.0)	2.02 (1.93, 2.12)	0.56 (0.52, 0.60)	5.22 (5.02, 5.40)
	Recovered	11,628	78.9 (78.6, 79.1)	– –	– –	– –
Outpatient office visit						
	Urgent care	801	9.6 (9.5, 9.8)	<0.2	<0.2	0.38 (0.36, 0.39)
	Emergency department	606	7.3 (7.2, 7.4)	0.89 (0.77, 1.03)	0.25 (0.21, 0.29)	3.23 (2.80, 3.82)
	Inpatient admission	335	4.0 (4.0, 4.1)	0.86 (0.71, 1.08)	0.23 (0.18, 0.28)	3.27 (2.62, 4.01)
	Recovered	6,573	79.0 (78.9, 79.2)	– –	– –	– –
Urgent care						
	Emergency department	856	6.7 (6.6, 6.8)	0.76 (0.75, 0.78)	0.23 (0.22, 0.23)	2.55 (2.48, 2.62)
	Inpatient admission	217	1.7 (1.7, 1.7)	0.42 (0.34, 0.54)	<0.2	1.42 (1.12, 1.82)
	Recovered	11,767	91.6 (91.5, 91.7)	– –	– –	– –
Emergency department						
	Inpatient admission	1,055	7.5 (7.4, 7.6)	0.69 (0.68, 0.70)	<0.2	2.37 (2.31, 2.44)
	Mechanical ventilation	8	0.1 (0.0, 0.1)	1.32 (0.55, 1.76)	0.80 (0.24, 1.31)	1.81 (1.08, 2.39)
	Death	69	0.5 (0.4, 0.6)	3.99 (2.88, 5.44)	1.29 (0.70, 2.02)	9.33 (7.09, 12.07)
	Recovered	12,984	91.9 (91.7, 92.1)	– –	– –	– –
Inpatient admission						
	Mechanical ventilation	232	5.1 (5.0, 5.2)	2.64 (2.51, 2.77)	0.79 (0.68, 0.89)	6.50 (5.94, 7.09)
	Death	293	6.5 (6.3, 6.6)	10.03 (9.85, 13.10)	5.26 (5.13, 8.80)	14.64 (14.37, 16.75)
	Recovered	4004	88.4 (88.2, 88.6)	– –	– –	– –
Mechanical ventilation						
	Death	112	40.7 (40.1, 41.4)	2.89 (2.73, 3.02)	0.80 (0.64, 0.94)	7.44 (6.55, 8.37)
	Recovered	163	59.3 (58.6, 59.9)	– –	– –	– –
*Influenza infections*						
Symptoms onset						
	Virtual care	1,545	8.1 (7.9, 8.2)	3.05 (3.00, 3.11)	1.81 (1.77, 1.86)	5.14 (5.03, 5.26)
	Outpatient office visit	2,415	12.6 (12.4, 12.9)	3.53 (3.46, 3.60)	2.12 (2.08, 2.17)	5.87 (5.74, 6.01)
	Urgent care	7,060	36.9 (36.4, 37.4)	2.99 (2.94, 3.05)	1.83 (1.79, 1.87)	4.90 (4.79, 5.01)
	Emergency department	5,468	28.6 (28.1, 29.0)	3.40 (3.34, 3.47)	2.04 (2.00, 2.09)	5.68 (5.55, 5.81)
	Inpatient admission	640	3.3 (3.3, 3.4)	4.61 (4.52, 4.70)	2.70 (2.64, 2.76)	7.86 (7.69, 8.04)
	Recovered	1,996	10.4 (10.3, 10.5)	– –	– –	– –
Receipt of test						
	Virtual care	1,396	6.8 (6.6, 6.9)	<0.2	<0.2	0.48 (0.47, 0.50)
	Outpatient office visit	2,400	11.6 (11.4, 11.8)	<0.2	<0.2	0.23 (0.23, 0.24)
	Urgent care	8,012	38.8 (38.3, 39.3)	<0.2	<0.2	<0.2
	Emergency department	6,436	31.2 (30.7, 31.7)	<0.2	<0.2	<0.2
	Inpatient admission	640	3.1 (3.0, 3.2)	<0.2	<0.2	<0.2
	Recovered	1,759	8.5 (8.4, 8.6)	– –	– –	– –
Virtual care						
	Outpatient office visit	456	12.4 (12.3, 12.6)	2.43 (2.35, 2.50)	0.72 (0.65, 0.78)	5.99 (5.64, 6.35)
	Urgent care	416	11.2 (11.1, 11.4)	0.70 (0.69, 0.71)	0.23 (0.23, 0.24)	2.12 (2.06, 2.16)
	Emergency department	426	11.7 (11.5, 11.9)	0.85 (0.74, 0.98)	0.27 (0.24, 0.29)	2.32 (2.18, 2.57)
	Inpatient admission	48	1.3 (1.0, 1.7)	1.79 (1.37, 2.38)	0.74 (0.56, 0.97)	3.58 (2.70, 4.69)
	Recovered	2,322	63.3 (62.8, 63.6)	– –	– –	– –
Outpatient office visit						
	Urgent care	647	14.0 (13.8, 14.2)	0.31 (0.30, 0.31)	<0.2	0.98 (0.95, 1.01)
	Emergency department	357	7.7 (7.6, 7.8)	1.17 (1.14, 1.19)	0.32 (0.31, 0.33)	3.23 (3.17, 3.29)
	Inpatient admission	102	2.2 (1.9, 2.7)	0.82 (0.55, 120)	<0.2	2.73 (2.07, 3.74)
	Recovered	3,522	76.1 (75.6, 76.6)	– –	– –	– –
Urgent care						
	Emergency department	674	7.3 (7.2, 7.4)	0.67 (0.65, 0.68)	<0.2	2.10 (2.04, 2.17)
	Inpatient admission	118	1.3 (1.3, 1.4)	0.52 (0.38, 0.73)	<0.2	1.82 (1.30, 2.46)
	Recovered	8,421	91.4 (91.3, 91.5)	– –	– –	– –
Emergency department						
	Inpatient admission	261	3.5 (3.5, 3.6)	0.67 (0.55, 0.83)	0.21 (0.17, 0.26)	2.15 (1.75, 2.68)
	Mechanical ventilation	8	0.1 (0.1, 0.2)	0.73 (0.24, 2.31)	0.24 (0.07, 0.77)	2.28 (0.67, 8.38)
	Death	9	0.1 (0.1, 0.2)	10.66 (5.07, 13.52)	6.16 (2.21, 10.12)	15.08 (9.61, 16.85)
	Recovered	7,146	96.3 (96.1, 96.4)	– –	– –	– –
Inpatient admission						
	Mechanical ventilation	70	6.3 (4.9, 7.9)	0.83 (0.54, 1.26)	0.25 (0.17, 0.38)	2.73 (1.82, 4.14)
	Death	36	3.2 (2.3, 4.3)	8.61 (6.08, 10.50)	4.84 (3.12, 6.70)	12.40 (10.38, 13.97)
	Recovered	1006	90.5 (88.6, 92.1)	– –	– –	– –
Mechanical ventilation						
	Death	23	27.4 (19.2, 38.3)	2.97 (1.40, 5.32)	0.81 (0.22, 1.88)	7.73 (4.53, 13.11)
	Recovered	61	72.6 (61.7, 80.8)	– –	– –	– –
*RSV infections*						
Symptoms onset						
	Virtual care	97	6.1 (5.6, 6.6)	5.39 (5.19, 5.58)	3.14 (2.80, 3.42)	8.55 (8.01, 9.11)
	Outpatient office visit	203	12.7 (12.2, 13.1)	4.58 (4.40, 4.74)	2.68 (2.46, 2.88)	7.24 (6.85, 7.61)
	Urgent care	170	10.6 (10.2, 11.0)	4.07 (3.98, 4.18)	2.45 (2.39, 2.52)	6.76 (6.59, 6.95)
	Emergency department	606	37.8 (37.2, 38.4)	4.18 (4.08, 4.28)	2.60 (2.53, 2.68)	6.72 (6.54, 6.91)
	Inpatient admission	299	18.7 (18.1, 19.2)	4.74 (4.63, 4.85)	3.00 (2.92, 3.09)	7.48 (7.29, 7.69)
	Recovered	227	14.2 (12.6, 15.9)	– –	– –	– –
Receipt of test						
	Virtual care	98	5.9 (4.9, 7.1)	2.13 (2.04, 2.21)	0.52 (0.41, 0.61)	5.93 (5.13, 6.73)
	Outpatient office visit	300	18.1 (17.6, 18.6)	0.28 (0.23, 0.34)	<0.2	0.90 (0.74, 1.09)
	Urgent care	153	9.3 (8.5, 10.0)	<0.2	<0.2	<0.2
	Emergency department	238	36.2 (35.7, 36.7)	<0.2	<0.2	<0.2
	Inpatient admission	599	14.4 (13.9, 14.9)	<0.2	<0.2	<0.2
	Recovered	265	16.0 (14.4, 17.8)	– –	– –	– –
Virtual care						
	Outpatient office visit	70	18.0 (14.6, 22.6)	3.00 (2.85, 3.10)	0.91 (0.68, 1.10)	7.27 (6.22, 8.28)
	Urgent care	20	5.1 (3.3, 7.7)	0.45 (0.20, 0.91)	0.14 (0.06, 0.33)	1.47 (0.64, 3.56)
	Emergency department	59	15.2 (11.9, 19.1)	1.19 (0.78, 1.80)	0.35 (0.21, 0.57)	3.15 (2.29, 4.33)
	Inpatient admission	17	4.4 (2.7, 7.1)	1.28 (0.80, 2.01)	0.53 (0.33, 0.89)	2.57 (1.61, 4.29)
	Recovered	223	57.3 (52.2, 61.6)	– –	– –	– –
Outpatient office visit						
	Urgent care	48	7.1 (5.3, 9.4)	1.76 (0.99, 2.74)	0.37 (0.15, 0.79)	5.27 (3.40, 8.11)
	Emergency department	112	16.6 (14.0, 19.5)	0.78 (0.57, 1.07)	0.24 (0.17, 0.34)	2.52 (1.80, 3.53)
	Inpatient admission	76	11.3 (9.1, 13.9)	0.48 (0.32, 0.74)	<0.2	1.72 (1.15, 2.59)
	Recovered	438	65.0 (61.5, 68.4)	– –	– –	– –
Urgent care						
	Emergency department	101	29.4 (24.8, 34.6)	0.55 (0.41, 0.74)	0.19 (0.14, 0.26)	1.58 (1.16, 2.12)
	Inpatient admission	60	17.5 (13.8, 21.8)	0.36 (0.25, 0.53)	<0.2	0.99 (0.69, 1.43)
	Recovered	182	53.1 (47.7, 58.4)	– –	– –	– –
Emergency department						
	Inpatient admission	141	16.2 (15.9, 16.4)	0.62 (0.48, 0.82)	0.22 (0.17, 0.28)	1.80 (1.37, 2.35)
	Mechanical ventilation	1	0.1 (0.0, 0.8)	0.09 (0.00, 0.15)	<0.2	0.11 (0.00, 0.20)
	Death	4	0.5 (0.2, 1.2)	5.72 (2.10, 15.00)	2.37 (0.88, 7.35)	11.44 (4.24, 35.41)
	Recovered	725	83.2 (82.3, 83.7)	– –	– –	– –
Inpatient admission						
	Mechanical ventilation	35	6.5 (4.6, 8.9)	2.07 (1.23, 3.09)	0.66 (0.29, 1.26)	4.84 (3.23, 7.12)
	Death	9	1.7 (0.8, 3.2)	8.51 (4.81, 10.14)	5.48 (1.99, 7.97)	11.20 (7.92, 12.05)
	Recovered	496	91.9 (89.1, 93.9)	– –	– –	– –
Mechanical ventilation						
	Death	5	12.8 (5.3, 27.7)	6.07 (2.24, 7.10)	4.17 (0.99, 5.91)	7.68 (4.42, 8.14)
	Recovered	34	87.2 (72.3, 94.7)	– –	– –	– –

We indicate values as <0.2 where maximum likelihood estimates and accompanying confidence limits fell below 0.2 days, which arose in scenarios where a vast majority of transitions along the indicated path were observed occurring on the same day. We report estimates from best-fitting distributions, based on models yielding the minimum AIC score; we indicate these distributions and their parameter estimates in S4 Table. Estimates and parameters of the same progressions using a longer follow-up period of 60 days are available in S6 Table and S7 Table in S1 File.

**Fig 3:**
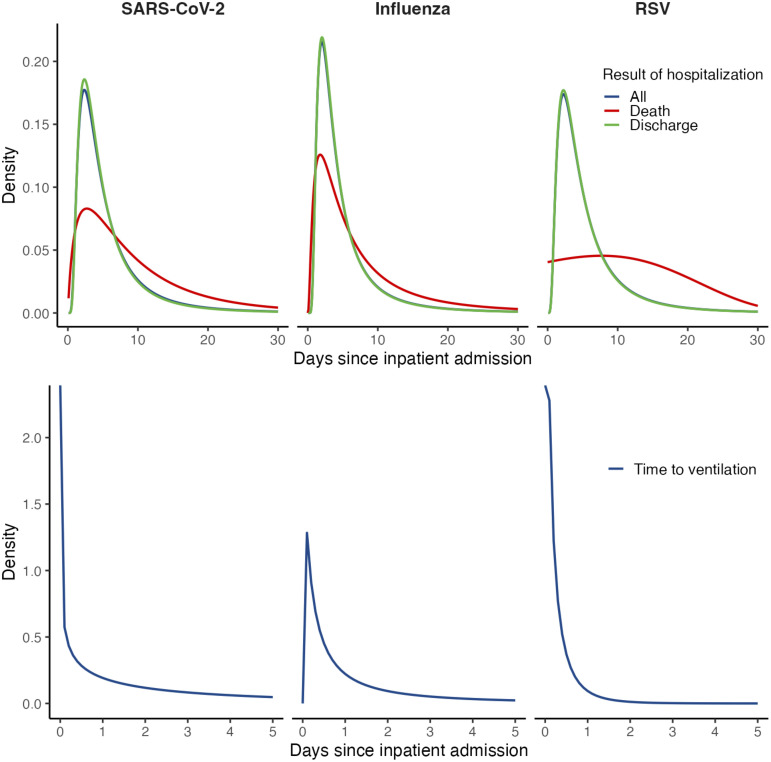
Durations of hospital stay. (*Top row*) We plot distributions from best-fitting models for durations of hospital stay, overall and stratified according to clinical outcome. (*Bottom row*) We plot distributions of time from inpatient admission to initiation of mechanical ventilation.

Median duration of inpatient stay for SARS-CoV-2 infections was 4.2 days (IQR: 2.6-7.3); median time to discharge was 4.1 days (IQR: 2.6-6.9) for patients who were discharged alive (Fig 3; S8 Table in S1 File). In the 20 days following inpatient admission for SARS-CoV-2 infections, 5.1% of patients required mechanical ventilation after a median 2.6 days (IQR: 0.8-6.5; Table 2). Median time to in-hospital death was 7.3 days (IQR: 3.7-13.3). Accounting for both in-hospital and out-of-hospital mortality, the 60-day risk of death after inpatient admission was 14.1%, with 11.3% of admitted patients dying without proceeding to mechanical ventilation, and 50.2% dying after initiating mechanical ventilation (Table 3; S6 Table in S1 File).

**Table 3:** Care utilization pathways at each acuity threshold for each infecting virus.

Originating state	Highest-acuity outcome	Probability of progression	Time to progression along indicated transition pathway, days (95% CI)		
		*Proportion, % (95% confidence interval)*	*Median*	*25%ile*	*75%ile*
*SARS-CoV-2 infections*					
Symptoms onset					
	Virtual care (*or higher*)	70.0 (69.6, 70.4)	3.90 (3.86, 3.94)	2.14 (2.13, 2.16)	7.11 (7.04, 7.18)
	Outpatient office visit (*or higher*)	56.3 (55.9, 56.7)	4.17 (4.09, 4.24)	2.22 (2.18, 2.24)	7.82 (7.65, 7.99)
	Urgent care (*or higher*)	48.3 (47.9, 48.7)	4.04 (3.99, 4.08)	2.18 (2.16, 2.20)	7.47 (7.38, 7.56)
	Emergency department (*or higher*)	27.9 (27.5, 28.3)	4.49 (4.42, 4.57)	2.42 (2.37, 2.43)	8.51 (8.37, 8.65)
	Inpatient admission (*or higher*)	7.9 (7.7, 8.1)	6.83 (6.64, 7.04)	3.55 (3.45, 3.67)	13.17 (12.70, 13.57)
	Mechanical ventilation (*or higher*)	1.7 (1.6, 1.8)	22.82 (21.67, 23.99)	11.95 (11.30, 12.73)	34.78 (33.40, 36.02)
	Death	1.5 (1.0, 1.8)	26.48 (22.10, 32.96)	15.16 (14.00, 15.81)	37.67 (30.50, 50.98)
Receipt of test					
	Virtual care (*or higher*)	65.3 (65.0, 65.6)	0.21 (0.20, 0.21)	<0.2	0.59 (0.58, 0.60)
	Outpatient office visit (*or higher*)	54.0 (53.4, 54.7)	0.17 (0.17, 0.18)	<0.2	0.49 (0.47, 0.50)
	Urgent care (*or higher*)	46.9 (46.3, 47.4)	0.15 (0.15, 0.16)	<0.2	0.37 (0.36, 0.38)
	Emergency department (*or higher*)	28.0 (27.7, 28.4)	0.16 (0.16, 0.16)	<0.2	0.40 (0.39, 0.41)
	Inpatient admission (*or higher*)	7.3 (7.1, 7.4)	0.36 (0.35, 0.37)	<0.2	1.52 (1.47, 1.58)
	Mechanical ventilation (*or higher*)	1.6 (1.0, 1.7)	12.92 (12.50, 13.33)	5.01 (4.85, 5.25)	27.02 (26.30, 27.74)
	Death	1.4 (1.0, 1.4)	19.48 (16.80, 23.40)	9.36 (8.88, 9.64)	32.17 (26.20, 41.62)
Virtual care					
	Outpatient office visit (*or higher*)	27.1 (26.0, 27.5)	4.28 (4.05, 4.50)	0.78 (0.70, 0.83)	13.94 (13.40, 14.45)
	Urgent care (*or higher*)	18.2 (17.0, 18.5)	1.96 (1.89, 2.02)	0.45 (0.43, 0.46)	7.78 (7.44, 8.14)
	Emergency department (*or higher*)	12.2 (12.0, 12.3)	1.91 (1.75, 2.09)	0.45 (0.44, 0.47)	7.38 (6.93, 7.91)
	Inpatient admission (*or higher*)	4.3 (4.0, 4.4)	6.26 (5.97, 6.56)	1.54 (1.51, 1.59)	17.19 (16.60, 17.78)
	Mechanical ventilation (*or higher*)	1.1 (1.0, 1.1)	21.58 (17.50, 26.38)	10.72 (8.80, 12.39)	34.46 (27.8, 63.00)
	Death	0.9 (0.0, 0.9)	23.67 (19.00, 30.08)	12.48 (12.10, 14.19)	35.86 (29.20, 80.40)
Outpatient office visit					
	Urgent care (*or higher*)	26.0 (20.0, 26.4)	1.10 (1.09, 1.12)	0.23 (0.21, 0.23)	5.42 (5.28, 5.55)
	Emergency department (*or higher*)	16.6 (16.0, 16.8)	2.41 (2.11, 2.76)	0.49 (0.45, 0.52)	9.35 (8.74, 10.31)
	Inpatient admission (*or higher*)	8.8 (8.0, 9.0)	4.88 (4.67, 5.10)	0.97 (0.90, 0.97)	15.15 (14.6, 15.66)
	Mechanical ventilation (*or higher*)	3.5 (3.0, 3.5)	11.69 (11.30, 11.97)	4.26 (4.13, 4.72)	25.43 (23.8, 27)
	Death	2.9 (2.0, 3.0)	18.50 (15.40, 22.05)	8.68 (7.61, 10.15)	31.32 (26, 38.78)
Urgent care					
	Emergency department (*or higher*)	9.4 (9.2, 9.5)	1.03 (1.01, 1.05)	0.25 (0.25, 0.25)	4.24 (4.12, 4.38)
	Inpatient admission (*or higher*)	2.7 (2.0, 2.7)	1.13 (0.90, 1.44)	0.24 (0.20, 0.30)	5.28 (4.21, 6.74)
	Mechanical ventilation (*or higher*)	0.5 (0.0, 0.7)	20.08 (14.80, 25.82)	9.75 (7.14, 13.52)	32.79 (26.10, 39.37)
	Death	0.4 (0.0, 0.5)	24.35 (17.50, 30.59)	13.36 (10.80, 17.87)	35.73 (28.50, 43.37)
Emergency department					
	Inpatient admission (*or higher*)	9.5 (9.0, 9.7)	1.39 (1.36, 1.41)	0.31 (0.30, 0.32)	6.16 (5.96, 6.35)
	Mechanical ventilation (*or higher*)	2.8 (2.0, 2.9)	17.48 (15.20, 20.35)	8.04 (7.63, 9.20)	30.28 (26.10, 36.73)
	Death	2.5 (2.0, 2.5)	19.40 (16.50, 22.48)	9.31 (8.15, 10.82)	32.09 (27.20, 38.70)
Inpatient admission					
	Mechanical ventilation (*or higher*)	17.2 (17.0, 17.4)	12.40 (11.80, 13.11)	4.06 (4.00, 4.18)	25.24 (24.20, 26.81)
	Death	14.1 (13.0, 14.2)	15.70 (15.30, 16.11)	8.03 (7.85, 8.18)	27.31 (26.70, 27.88)
Mechanical ventilation					
	Death	50.2 (44.3, 55.6)	5.24 (4.95, 5.48)	1.25 (1.00, 1.47)	14.66 (12.86, 21.64)
*Influenza infections*					
Symptoms onset					
	Virtual care (*or higher*)	93.1 (92.7, 93.4)	3.39 (3.30, 3.47)	1.98 (1.94, 2.03)	5.78 (5.61, 5.95)
	Outpatient office visit (*or higher*)	87.7 (87.2, 88.2)	3.52 (3.43, 3.61)	2.05 (2.00, 2.07)	6.05 (5.89, 6.23)
	Urgent care (*or higher*)	77.4 (76.8, 77.9)	3.55 (3.50, 3.60)	2.05 (2.04, 2.08)	6.14 (6.05, 6.22)
	Emergency department (*or higher*)	38.5 (38.0, 39.0)	4.00 (3.92, 4.08)	2.30 (2.29, 2.34)	7.12 (6.96, 7.28)
	Inpatient admission (*or higher*)	5.8 (5.7, 5.9)	6.60 (6.49, 6.73)	3.52 (3.45, 3.56)	12.14 (11.88, 12.40)
	Mechanical ventilation (*or higher*)	0.9 (0.8, 0.9)	15.35 (15.05, 15.68)	7.69 (7.55, 7.87)	26.29 (25.80, 26.88)
	Death	0.5 (0.5, 0.6)	22.64 (22.10, 23.21)	13.92 (13.74, 14.10)	33.33 (32.54, 34.17)
Receipt of test					
	Virtual care (*or higher*)	92.1 (91.9, 92.2)	<0.2	<0.2	0.21 (0.20, 0.21)
	Outpatient office visit (*or higher*)	87.9 (87.7, 88.1)	<0.2	<0.2	0.20 (0.20, 0.21)
	Urgent care (*or higher*)	78.5 (78.2, 78.8)	<0.2	<0.2	0.20 (0.20, 0.20)
	Emergency department (*or higher*)	39.0 (38.6, 39.4)	<0.2	<0.2	0.25 (0.24, 0.25)
	Inpatient admission (*or higher*)	5.3 (5.2, 5.3)	0.31 (0.30, 0.32)	<0.2	1.18 (1.14, 1.21)
	Mechanical ventilation (*or higher*)	0.9 (0.8, 0.9)	7.68 (7.35, 7.95)	2.03 (1.78, 2.20)	20.26 (18.40, 22.38)
	Death	0.6 (0.6 0.6)	14.06 (13.70, 14.39)	5.84 (5.72, 5.90)	28.13 (27.50, 28.77)
Virtual care					
	Outpatient office visit (*or higher*)	42.6 (42.2, 43.0)	1.62 (1.59, 1.65)	0.42 (0.41, 0.43)	6.26 (6.09, 6.42)
	Urgent care (*or higher*)	29.6 (29.3, 30.0)	1.23 (1.21, 1.26)	0.33 (0.32, 0.34)	4.63 (4.51, 4.77)
	Emergency department (*or higher*)	16.9 (16.6, 17.2)	1.40 (1.36, 1.43)	0.38 (0.38, 0.39)	5.13 (4.94, 5.33)
	Inpatient admission (*or higher*)	3.2 (3.2, 3.3)	6.58 (6.26, 6.88)	1.73 (1.39, 2.07)	17.47 (15.40, 19.65)
	Mechanical ventilation (*or higher*)	0.8 (0.6, 1.1)	13.53 (9.78, 18.79)	5.62 (3.38, 7.34)	27.06 (18.81, 39.06)
	Death	0.6 (0.4, 0.9)	16.75 (15.74, 17.34)	8.99 (6.95, 10.40)	28.15 (24.41, 31.63)
Outpatient office visit					
	Urgent care (*or higher*)	27.8 (27.5, 28.1)	0.81 (0.80, 0.83)	0.18 (0.18, 0.19)	3.66 (3.56, 3.77)
	Emergency department (*or higher*)	14.3 (14.0, 14.6)	1.30 (1.27, 1.33)	0.32 (0.31, 0.32)	5.31 (511, 5.51)
	Inpatient admission (*or higher*)	5.0 (5.0, 5.1)	3.36 (3.20, 3.50)	0.53 (0.44, 0.65)	11.84 (10.50, 13.33)
	Mechanical ventilation (*or higher*)	1.2 (0.9, 1.5)	7.54 (4.71, 11.64)	1.77 (1.02, 4.25)	21.29 (14.60, 29.94)
	Death	0.8 (0.5, 1.1)	16.07 (9.40, 28.44)	6.32 (5.47, 6.98)	32.17 (17.27, 58.71)
Urgent care					
	Emergency department (*or higher*)	9.2 (9.0, 9.3)	0.82 (0.81, 0.84)	0.22 (0.21, 0.22)	3.04 (2.93, 3.14)
	Inpatient admission (*or higher*)	1.9 (1.9, 1.9)	1.47 (1.06, 2.04)	0.25 (0.19, 0.30)	5.79 (4.40, 7.67)
	Mechanical ventilation (*or higher*)	0.2 (0.2, 0.4)	12.22 (8.00, 18.27)	5.07 (4.07, 7.87)	24.44 (15.90,36.21)
	Death	0.1 (0.1, 0.2)	16.08 (8.70, 28.85)	6.67 (3.62, 9.82)	32.16 (17.00, 51.19)
Emergency department					
	Inpatient admission (*or higher*)	4.5 (4.5, 4.6)	1.43 (1.13, 1.79)	0.33 (0.25, 0.41)	6.24 (5.02, 7.83)
	Mechanical ventilation (*or higher*)	1.0 (0.8, 1.3)	12.84 (9.30, 17.10)	4.61 (2.50, 5.05)	28.17 (21.80, 36.37)
	Death	0.7 (0.5, 0.9)	22.95 (16.50, 29.65)	11.73 (10.20, 15.45)	35.71 (28.40, 43.84)
Inpatient admission					
	Mechanical ventilation (*or higher*)	12.3 (12.0, 12.6)	5.56 (5.22, 5.89)	1.22 (1.01, 1.39)	16.31 (14.10, 18.68)
	Death	7.9 (6.5, 9.7)	13.47 (10.90, 16.40)	5.59 (5.22, 6.51)	26.95 (21.60, 33.06)
Mechanical ventilation					
	Death	34.5 (25.4, 45.7)	5.52 (2.87, 9.58)	1.35 (0.40, 3.19)	15.28 (9.02, 24.68)
*RSV infections*					
Symptoms onset					
	Virtual care (*or higher*)	92.5 (91.1, 93.6)	4.69 (4.51, 4.87)	2.78 (2.72, 2.86)	7.93 (7.59, 8.28)
	Outpatient office visit (*or higher*)	90.3 (88.6, 91.7)	4.79 (4.60, 5.00)	2.83 (2.75, 2.86)	8.10 (7.77, 8.49)
	Urgent care (*or higher*)	81.7 (79.6, 83.4)	5.00 (4.78, 5.23)	2.97 (2.89, 3.02)	8.42 (8.05, 8.82)
	Emergency department (*or higher*)	74.1 (72.0, 76.1)	5.23 (5.02, 5.46)	3.12 (3.06, 3.28)	8.76 (8.36, 9.21)
	Inpatient admission (*or higher*)	33.8 (31.8, 36.2)	6.36 (5.95, 6.79)	3.80 (3.61, 4.11)	10.63 (9.91, 11.46)
	Mechanical ventilation (*or higher*)	4.2 (3.3, 5.3)	13.39 (11.06, 16.20)	7.74 (6.17, 8.10)	23.15 (18.40, 28.62)
	Death	2.1 (1.5, 2.9)	23.73 (19.52, 29.63)	14.91 (13.20, 6.67)	34.22 (28.70, 40.63)
Receipt of test					
	Virtual care (*or higher*)	90.7 (89.3, 92.0)	<0.2	<0.2	0.33 (0.32, 0.34)
	Outpatient office visit (*or higher*)	89.2 (89.1, 89.4)	<0.2	<0.2	0.31 (0.30, 0.31)
	Urgent care (*or higher*)	79.4 (79.1, 79.6)	<0.2	<0.2	0.25 (0.24, 0.25)
	Emergency department (*or higher*)	71.9 (71.5, 72.2)	<0.2	<0.2	0.27 (0.26, 0.28)
	Inpatient admission (*or higher*)	31.5 (31.0, 32.0)	0.24 (0.23, 0.24)	<0.2	0.71 (0.69, 0.74)
	Mechanical ventilation (*or higher*)	4.2 (3.4, 5.3)	6.4 (4.29, 9.1)	1.75 (1.01, 2.27)	16.56 (12.10, 22.24)
	Death	2.2 (1.6, 2.9)	13.52 (9.7, 18.73)	5.62 (4.64, 7.11)	27.05 (19.00, 37.36)
Virtual care					
	Outpatient office visit (*or higher*)	54.6 (49.5, 59.5)	3.64 (2.77, 4.86)	0.74 (0.54, 0.96)	12.73 (10.10, 16.22)
	Urgent care (*or higher*)	35.4 (34.9, 35.9)	1.71 (1.22, 2.45)	0.44 (0.43, 0.60)	6.66 (4.73, 9.26)
	Emergency department (*or higher*)	30.0 (29.5, 30.5)	1.8 (1.28, 2.59)	0.49 (0.39, 0.56)	6.67 (4.64, 9.35)
	Inpatient admission (*or higher*)	13.6 (10.5, 17.3)	3.3 (1.97, 5.15)	0.95 (0.55, 1.34)	11.50 (6.67, 20.69)
	Mechanical ventilation (*or higher*)	1.8 (0.9, 3.7)	16.04 (7.00, 23.32)	6.66 (4.45, 10.19)	32.08 (15.10, 47.16)
	Death	1.0 (0.4, 2.7)	17.34 (6.10, 25.69)	7.20 (4.24, 10.22)	34.69 (13.20, 56.05)
Outpatient office visit					
	Urgent care (*or higher*)	40.2 (39.9, 40.6)	1.08 (0.85, 1.39)	0.26 (0.21, 0.33)	4.50 (3.46, 5.79)
	Emergency department (*or higher*)	35.0 (34.6, 35.4)	1.03 (0.79, 1.32)	0.25 (0.20, 0.30)	4.28 (3.26, 5.67)
	Inpatient admission (*or higher*)	16.8 (16.0, 17.5)	1.24 (0.84, 1.81)	0.27 (0.21, 0.32)	5.64 (3.84, 8.34)
	Mechanical ventilation (*or higher*)	4.7 (3.3, 6.6)	8.48 (4.99, 13.55)	2.47 (1.86, 3.47)	21.09 (13.90, 32.42)
	Death	2.9 (1.9, 4.4)	17.12 (11.10, 25.90)	7.11 (5.74, 8.70)	34.24 (22.60, 41.47)
Urgent care					
	Emergency department (*or higher*)	48.3 (47.7, 48.8)	0.53 (0.42, 0.71)	<0.2	1.68 (1.30, 2.12)
	Inpatient admission (*or higher*)	22.4 (18.1, 27.1)	0.52 (0.36, 0.76)	<0.2	1.62 (1.08, 2.45)
	Mechanical ventilation (*or higher*)	1.5 (0.6, 3.4)	9.15 (3.61, 22.13)	3.80 (1.99, 6.12)	18.29 (7.71, 33.37)
	Death				
Emergency department					
	Inpatient admission (*or higher*)	18.4 (15.9, 21.1)	0.95 (0.70, 1.29)	0.26 (0.21, 0.26)	3.49 (2.63, 4.61)
	Mechanical ventilation (*or higher*)	2.2 (1.4, 3.4)	10.65 (6.60, 16.67)	4.42 (3.61, 5.03)	21.31 (13.70, 34.45)
	Death	1.3 (0.7, 2.3)	11.21 (6.20, 19.98)	4.66 (2.54, 8.24)	22.43 (12.40, 38.45)
Inpatient admission					
	Mechanical ventilation (*or higher*)	10.9 (8.5, 13.8)	5.90 (3.91, 8.46)	1.64 (0.58, 2.75)	15.07 (10.60, 20.68)
	Death	5.0 (3.5, 7.2)	17.77 (16.80, 28.52)	9.69 (8.93, 11.33)	29.55 (26.30, 32.96)
Mechanical ventilation					
	Death	20.5 (10.2, 37.1)	11.96 (5.77, 22.86)	4.96 (2.43, 10.48)	23.91 (11.71, 50.51)

We indicate values as <0.2 where maximum likelihood estimates and accompanying confidence limits fell below 0.2 days, which arose in scenarios where a vast majority of transitions along the indicated path were observed occurring on the same day. We report estimates from best-fitting distributions, based on models yielding the minimum AIC score; we indicate these distributions and their parameters parameter in S5 Table in S1 File.

### Care pathways for influenza and RSV

Median times from symptoms onset to testing were 3.4 and 5.6 days for influenza and RSV, respectively, corresponding to differences in the clinical care settings at which testing most frequently occurred for each pathogen (**Fig 2**). The first clinical encounter occurred in urgent care for 36.7% of influenza cases, in emergency departments for 28.8% of cases, and in hospital settings for 3.4%; a greater proportion of confirmed RSV infections (18.7%) were first intercepted in hospital settings (**Table 2**).

Median durations of hospital stay for influenza cases and RSV cases were 4.0 days (IQR: 2.3-6.8) and 4.3 days (IQR: 2.5-7.4), respectively (S8 Table in S1 File). The 60-day risk of death after hospital admission was 7.9% among influenza cases and 5.0% among RSV cases (Table 3). Median time to in-hospital death following admission was 5.2 days (IQR: 2.6-10.5) among influenza cases and 11.3 days (IQR: 5.8-17.6) among RSV cases. In the 60 days following an inpatient admission, median times from admission to death were 17.5 days (IQR: 4.1-31.4) and 18.7 days (9.2-30.0) for influenza and RSV cases, respectively, who did not require mechanical ventilation, while median times from initiation of mechanical ventilation to death were 5.5 days (1.4-15.3) and 12.0 days (5.0-23.9) for influenza and RSV cases, respectively (S6 Table in S1 File).

### Care requirements for all observed infections

For SARS-CoV-2 infections, median times from symptoms onset to receipt of care at or above the virtual care, outpatient physician office, urgent care, or emergency department thresholds were in the range of 3.9-4.5 days (**Table 4**). Overall, 7.9% of all observed SARS-CoV-2 infections resulted in inpatient admission or death, occurring a median 6.8 days (IQR: 3.6-13.2) after symptoms onset. Progression to illness necessitating mechanical ventilation and death occurred markedly later in the course of illness (median 22.8 days [IQR: 12.0-34.8] and 26.2 days [IQR: 15.2-37.7] after symptoms onset, respectively) than initial inpatient admission.

**Table 4:** Observed proportions of cases attaining or exceeding each acuity threshold.

Infecting virus	Acuity threshold	Probability of progression	Time to progression, days (95% CI)		
		*Proportion, % (95% confidence interval)*	*Median*	*25%ile*	*75%ile*
SARS-CoV-2					
	Virtual care (*or higher*)	70.0 (69.6, 70.4)	3.90 (3.86, 3.94)	2.14 (2.13, 2.16)	7.11 (7.04, 7.18)
	Outpatient office visit (*or higher*)	56.3 (55.9, 56.7)	4.17 (4.09, 4.24)	2.22 (2.18, 2.24)	7.82 (7.65, 7.99)
	Urgent care (*or higher*)	48.3 (47.9, 48.7)	4.04 (3.99, 4.08)	2.18 (2.16, 2.20)	7.47 (7.38, 7.56)
	Emergency department (*or higher*)	27.9 (27.5, 28.3)	4.49 (4.42, 4.57)	2.42 (2.37, 2.43)	8.51 (8.37, 8.65)
	Inpatient admission (*or higher*)	7.9 (7.7, 8.1)	6.83 (6.64, 7.04)	3.55 (3.45, 3.67)	13.17 (12.70, 13.57)
	Mechanical ventilation (*or higher*)	1.7 (1.6, 1.8)	22.82 (21.67, 23.99)	11.95 (11.30, 12.73)	34.78 (33.40, 36.02)
	Death	1.3 (1.3, 1.4)	26.20 (24.93, 27.56)	15.16 (14.00, 15.81)	37.67 (30.50, 50.98)
Influenza					
	Virtual care (*or higher*)	93.1 (92.7, 93.4)	3.39 (3.30, 3.47)	1.98 (1.94, 2.03)	5.78 (5.61, 5.95)
	Outpatient office visit (*or higher*)	87.7 (87.2, 88.2)	3.52 (3.43, 3.61)	2.05 (2.00, 2.07)	6.05 (5.89, 6.23)
	Urgent care (*or higher*)	77.4 (76.8, 77.9)	3.55 (3.50, 3.60)	2.05 (2.04, 2.08)	6.14 (6.05, 6.22)
	Emergency department (*or higher*)	38.5 (38.0, 39.0)	4.00 (3.92, 4.08)	2.30 (2.29, 2.34)	7.12 (6.96, 7.28)
	Inpatient admission (*or higher*)	5.8 (5.7, 5.9)	6.60 (6.49, 6.73)	3.52 (3.45, 3.56)	12.14 (11.88, 12.40)
	Mechanical ventilation (*or higher*)	0.9 (0.8, 0.9)	15.35 (15.05, 15.68)	7.69 (7.55, 7.87)	26.29 (25.80, 26.88)
	Death	0.5 (0.5, 0.6)	22.64 (22.10, 23.21)	13.92 (13.74, 14.10)	33.33 (32.54, 34.17)
lRSV					
	Virtual care (*or higher*)	92.5 (91.1, 93.6)	4.69 (4.51, 4.87)	2.78 (2.72, 2.86)	7.93 (7.59, 8.28)
	Outpatient office visit (*or higher*)	90.3 (88.6, 91.7)	4.79 (4.60, 5.00)	2.83 (2.75, 2.86)	8.10 (7.77, 8.49)
	Urgent care (*or higher*)	81.7 (79.6, 83.4)	5.00 (4.78, 5.23)	2.97 (2.89, 3.02)	8.42 (8.05, 8.82)
	Emergency department (*or higher*)	74.1 (72.0, 76.1)	5.23 (5.02, 5.46)	3.12 (3.06, 3.28)	8.76 (8.36, 9.21)
	Inpatient admission (*or higher*)	33.8 (31.8, 36.2)	6.36 (5.95, 6.79)	3.80 (3.61, 4.11)	10.63 (9.91, 11.46)
	Mechanical ventilation (*or higher*)	4.2 (3.3, 5.3)	13.39 (11.06, 16.20)	7.74 (6.17, 8.10)	23.15 (18.40, 28.62)
	Death	2.1 (1.5, 2.9)	23.73 (19.52, 29.63)	14.91 (13.20, 16.67)	34.22 (28.70, 40.63)

We report estimates from best-fitting distributions, based on models yielding the minimum AIC score; we indicate these distributions and their parameter estimates in S4 Table in S1 File. Estimates and parameters of the same progressions using a longer follow-up period of 60 days are available in S6 Table and S7 Table in S1 File.

Nearly all influenza and RSV infections were linked to ARI diagnoses resulting from care appointments in any setting around the time of individuals’ first eligible positive test (93.1% and 92.5%, respectively; **Table 4**). Median times from symptoms onset to receipt of care at virtual, outpatient, urgent care, and emergency department or higher-acuity settings were 3.2, 3.5, 3.6, and 4.0 days, respectively, for influenza, and 4.7, 4.8, 5.0, and 5.2 days, respectively, for RSV (**Fig 2**). Median times from symptoms onset to inpatient admission, mechanical ventilation, and death were 6.4-6.6 days, 13.4-15.3 days, and 22.6-23.7 days, respectively.

### Associations of care trajectories with individual characteristics

**Fig 4:**
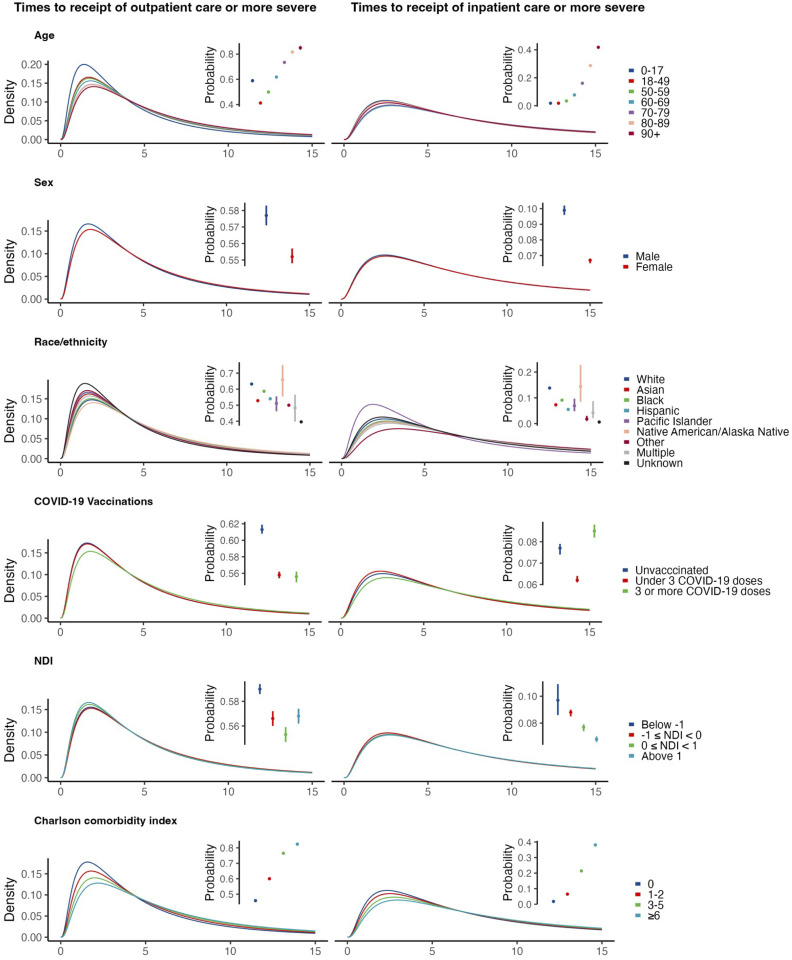
Probabilities and time-to-event distributions of reaching outpatient and inpatient acuity thresholds from symptom onset for illness associated with a SARS-CoV-2 infection across demographic subgroups. The best-fitting distributions for the symptom onset to outpatient/inpatient acuity threshold were used across covariates, but the corresponding location parameter was allowed to vary by subgroup. Probabilities of reaching acuity thresholds are available in S9–S13 Tables in S1 File.

The proportion of cases receiving care at each acuity level increased with older age for all infections; age differences were most pronounced for high-acuity outcomes (e.g., inpatient admission, mechanical ventilation, and mortality; **Fig 4**; S9 Table in S1 File). Median times from symptoms onset to receipt of care at or above the level of outpatient office visits increased with older age, spanning a difference of ~1 day between the ≤ 17 year and ≥90 year age groups for all three viral infections (3.3 vs. 4.7 days for SARS-CoV-2 infections, 3.2 vs. 4.4 days for influenza infections, and 4.0 vs. 4.8 days for RSV infections), although these differences across ages in times to event were attenuated for higher-acuity outcomes. Individuals with greater numbers of comorbid conditions also had higher chances of receiving care at each level of acuity and longer median times to presentation, for each virus (S10 Table)in S1 File).

Whereas a greater proportion of males than females with SARS-CoV-2 infection experienced high-acuity outcomes (e.g., 9.9% vs. 6.7% with inpatient admission or higher-acuity outcomes, 2.1% vs. 1.1% mortality; S11 Tablein S1 File), this pattern was less clearly apparent for influenza cases and was reversed for RSV. Times to each outcome were similar for male and female cases with each infection. With regard to individuals’ vaccination status and neighborhood deprivation index values, we did not identify patterns across pathogens or across outcomes with respect to any subgroup experiencing consistently higher or lower likelihood of progression, or consistently longer or shorter times to progression (S12 Table; S13 Table in S1 File).

The probability of in-hospital mortality for SARS-CoV-2 infections was higher among older adults compared to younger adults (11.0% at ages ≥90 years versus 2.1% in 18–49 year age group; S14 Table in S1 File). There were also significant differences across Charlson comorbidity subgroups, with 9.0% of cases with a score ≥6 experiencing in-hospital mortality associated with SARS-CoV-2 infection in comparison to 3.9% mortality among cases with a score of 0. This trend was also apparent in influenza infections (7.2% vs. 1.7%), although not in RSV infections.

Median durations of hospital stay were similar across groups for each infection. Males had a longer median time to in-hospital mortality than females for SARS-CoV-2 infections (8.5 vs. 6.8 days), but shorter times to mortality for influenza and RSV infections (4.9 vs. 5.7 days and 8.1 vs. 16.1 days, respectively; S14 Table in S1 File). We did not observe differences across subgroups with respect to vaccination status, race or ethnicity, or neighborhood deprivation index in the probability of in-hospital mortality or length of hospital stays.

### Github repository

In addition to the descriptive supplementary materials associated with this manuscript, we have created a Github repository containing parameter estimates for all analyses described (https://github.com/ntparker3/Resp_params). The repository includes four files for each pathogen (SARS-CoV-2, influenza, and RSV), contents of which are listed below:

1. Parameterized distributions and summary statistics of the proximal progression event occurring from each originating state (“first event”);
2. Parameterized distributions and summary statistics for individuals’ to risk of progression to or above each acuity threshold, from each originating state (“event or worse”);
3. Probabilities of progression to or above each acuity threshold, from each originating state, across subgroups of the specified individual-level covariates (“event or worse covariates”); and
4. Location parameters and median times to event for progression to outpatient (or higher-acuity) and inpatient (or higher-acuity) thresholds, from each originating state, across subgroups of the specified individual-level covariates (“covariate rates”).

## Discussion

Our analysis provides estimates of transition rates and probabilities for healthcare utilization due to the progression of ARIs associated with SARS-CoV-2, influenza, and RSV infections. These outputs aim to inform models anticipating resource needs for healthcare systems and public health stakeholders, drawing on real-world observations within a US managed care setting. In addition to presenting aggregated results for all cases infected with SARS-CoV-2, influenza, and RSV, we present stratified results for differing subgroups for which models may aim to generate predictions; these encompassed patient demographics (age, sex, race/ethnicity), comorbidity burden, prior vaccination, and community-level socioeconomic disadvantage. Among these characteristics, we identified the strongest evidence of differences in progression risk and times-to-event across age groups and comorbidity profiles. Our outputs fill frequently-described gaps in the data needed for application of viral respiratory infection models [24–26] and may inform future forecasting efforts tailored to US healthcare contexts, particularly those aiming to inform healthcare resource allocation [10,27,28].

Previous studies have reported widely varying estimates of times from symptoms onset to hospital admission for COVID-19 [29–32] and the duration of hospital stay among COVID-19 patients [13,14,33], with both parameters differing across settings and over time within settings in association with evolving clinical practices. Whereas numerous studies have monitored patients hospitalized with each virus [34,35], fewer have tracked outcomes longitudinally from early points in the disease course such as symptoms onset or receipt of care in virtual or ambulatory facilities. Within our study, only 4.7% of COVID-19 cases (7.9% of all COVID-19 cases who received care in any setting) were first intercepted at the point of hospital admission, while among influenza and RSV cases, 3.4% and 18.7%, respectively, were first seen in inpatient settings. These circumstances suggest that projecting outcomes among individuals receiving care in lower-acuity settings may help to refine forecasts of higher-acuity clinical care needs.

Application of our estimates to forecasting models requires several assumptions or considerations. First, we frame consecutive transitions between states as memoryless, consistent with modeling approaches where estimates from these analyses may be applied (e.g., Markov chain next-state transitions as well as cumulative probabilities of attaining each state overall and from preceding states). Second, our analyses are subset to individuals who ultimately received care including diagnostic testing: events preceding testing among individuals included in these analyses, particularly in low-acuity care settings, may not represent care utilization pathways among individuals who were ultimately never tested—a problem related to previously described biases affecting interval distribution estimation [8,36,37]. Furthermore, testing for influenza and RSV was more strictly limited to individuals who received care in outpatient or inpatient facilities, whereas SARS-CoV-2 tests were widely available across all care settings. In particular, RSV testing in adults is restricted to inpatient settings, with the majority of testing, and subsequent recorded cases, being conducted in children. Thus, differences in the overall proportions of SARS-CoV-2 infections, influenza infections, and RSV infections attaining each acuity threshold should not be interpreted as differences in the severity of disease caused by each infection. The clinical threshold associated with testing in our study population may also differ from that in other healthcare systems, geographic regions, or countries. Increased testing among individuals with less-severe disease would be expected to lower the proportion of episodes expected to progress to hospital admission or other high-acuity outcomes. As this circumstance could also occur through testing at earlier stages in individuals’ illness, such increases in testing for less-severe disease could lead to longer estimated times to progression. Last, associations of the studied covariates with the proportions of cases experiencing each outcome and with times-to-event should not be interpreted as causal. In some instances, observed patterns reflect previously reported independent associations, such as associations of older age and the presence of comorbidities with severe disease outcomes [38]. However, other findings, such as the lack of association of prior vaccination with protection against severe outcomes, echo previous evidence of higher uptake of vaccines against COVID-19, seasonal influenza, and RSV among individuals at greatest risk [39–41]. These analyses aim to inform prediction even if they lack direct causal interpretation.

Our analysis has at least 7 limitations. First, KPSC represents a single healthcare system. While strengths include the integration of care delivery and data capture across outpatient and inpatient settings, the large enrollee population, and its racial/ethnic and socioeconomic diversity (13), it remains important to note that care utilization and delivery pathways may not be generalizable to all settings. Estimation of similar parameters in other US populations remains an important objective. Second, aiming to enable the broad application of our estimates, we fit parametric distributions to times-to-event that may not perfectly represent underlying processes. For this reason, we supply best-fitting parameter estimates for 6 different distributions for all times-to-event. As AIC may not adequately penalize overfitting, or multiple distributions may provide similar fit to observed data, practitioners should consider mechanistic interpretations as well as underlying assumptions of differing distribution in choosing which may be the most appropriate to modeling applications. Third, major SARS-CoV-2 variants (e.g., XBB, BA.2.86/JN.1, and KP.2) and seasonal influenza lineages (e.g., A(H1N1)pdm09, A(H3N2), B(Victoria)) circulating during the study period may not generalize to lineages circulating during future years. Fourth, the reliability of self-reported and physician-recorded symptoms onset dates may be imperfect, as signified by patterns such as heaping of times from symptoms onset around 7 days and 14 days before testing and other healthcare encounters [42]. Fifth, recovery was not explicitly recorded as a clinical outcome, necessitating censoring of observation periods without future healthcare encounters. Sixth, while restricting progression events to healthcare encounters where ARI diagnoses were assigned was anticipated to reduce misclassification, coding practices (e.g., carry-forward of diagnosis codes) may lead to misclassification of some encounters. Cessation of healthcare facility-based SARS-CoV-2 screening by the time of our study was further anticipated to mitigate risks of misclassifying healthcare encounters “with” or “for” COVID-19. Last, our analyses preceded the widespread implementation of RSV vaccines among pregnant mothers and older adults, which may alter RSV-related healthcare utilization in future seasons for population groups at the highest risk of severe disease.

These limitations notwithstanding, our analyses provide a useful entry point for modeling real-world trajectories of healthcare needs associated with SARS-CoV-2, influenza, and RSV infections. Extensive ARI-associated healthcare utilization in virtual, outpatient, and urgent care settings among persons ultimately hospitalized suggests monitoring of lower-acuity healthcare utilization may help to inform near-term hospital capacity requirements. Incorporating data on lower-acuity care delivery settings into public health surveillance and reporting thus merits consideration. Similar analyses in other geographic settings or other healthcare systems, and continued updating of parameters we report to accommodate changes in viral epidemiology or healthcare delivery practices, may improve the reliability of forecasting models for SARS-CoV-2, influenza, and RSV.

## Supporting information

S1 File Supporting information, including S1 Table (Acute respiratory illness diagnosis codes), S2 Table (Counts of patients with comorbidities included in Charlson comorbidity index), S3 Table (Individual characteristics by infecting virus and severity threshold reached); S4 Table (Best-fitting distributions for care utilization pathways for all infections using a 20-day follow-up period); S5 Table (Best-fitting distributions for care utilization pathways at each acuity threshold using a 60-day follow-up period); S6 Tale (Care utilization pathways associated with each infecting virus using a follow-up period of 60 days); S7 Table (Best-fitting distributions for care utilization pathways for all infections using a 60-day follow-up period); S8 Table (Hospital length of stay estimates for admissions leading to discharge or mortality); S9 Table (Proportions of cases attaining or exceeding each acuity threshold, by age); S10 Table (Proportions of cases attaining or exceeding each acuity threshold, by Charlson comorbidity index values); S11 Table (Proportions of cases attaining or exceeding each acuity threshold, by sex); S12 Table (Proportions of cases attaining or exceeding each acuity threshold, by neighborhood deprivation index); S13 Table (Proportions of cases attaining or exceeding each acuity threshold, by vaccination status); S14 Table (Stratified hospital length of stay estimates for admissions associated with each viral infection and leading to discharge or mortality); S1 Fig (Healthcare utilization cascade); S2 Fig (Study flowchart). (ZIP)
