# Supplementary material for: Clinical progression parameters associated with SARS-CoV-2, influenza, and respiratory syncytial virus infections in a large US integrated healthcare population
Source: PLoS Comput Biol. 2025 Nov 19;21(11):e1013723. doi: 10.1371/journal.pcbi.1013723 (PMC12643285; doi:10.1371/journal.pcbi.1013723)
Supplement: S1 File — (ZIP) [file pcbi.1013723.s001.zip › S1 File/S10_Table.pdf]

**S10 Table: Proportions of cases attaining or exceeding each acuity threshold, by Charlson comorbidity index values.**

| Acuity threshold | Stratum | SARS-CoV-2 infections | | Influenza infections | | RSV infections | |
|---|---|---|---|---|---|---|---|
| | | *Proportion, % (95% CI)* | *Median time from symptoms onset to event, days (95% CI)* | *Proportion, % (95% CI)* | *Median time from symptoms onset to event, days (95% CI)* | *Proportion, % (95% CI)* | *Median time from symptoms onset to event, days (95% CI)* |
| Virtual care (or higher) | | | | | | | |
| | 0 | 61.6 (61.2, 62.0) | 3.54 (3.21, 3.87) | 92.4 (92.2, 92.5) | 3.15 (2.69, 3.65) | 90.6 (88.5, 92.4) | 3.96 (2.74, 5.89) |
| | 1-2 | 74.9 (74.4, 75.3) | 3.95 (3.43, 4.48) | 93.4 (93.2, 93.5) | 3.54 (2.91, 4.36) | 93.0 (90.0, 95.7) | 4.88 (2.78, 8.64) |
| | 3-5 | 85.5 (85.2, 85.8) | 4.48 (3.92, 5.11) | 96.8 (95.8, 97.6) | 4.34 (3.53, 5.42) | 95.6 (92.2, 97.5) | 5.78 (3.33, 9.61) |
| | ≥6 | 88.6 (88.3, 88.8) | 5.02 (4.40, 5.73) | 96.0 (94.3, 97.2) | 5.07 (4.12, 6.24) | 94.0 (89.0, 96.5) | 6.32 (3.68, 11.00) |
| Outpatient office visit (or higher) | | | | | | | |
| | 0 | 45.8 (45.4, 46.3) | 3.72 (3.42, 4.02) | 86.4 (86.1, 86.6) | 3.25 (2.80, 3.74) | 87.8 (85.3, 89.9) | 4.06 (2.78, 5.89) |
| | 1-2 | 59.4 (60.5, 60.5) | 4.23 (3.75, 4.77) | 88.1 (87.8, 88.4) | 3.70 (3.03, 4.55) | 91.4 (88.1, 93.9) | 4.92 (2.91, 8.13) |
| | 3-5 | 76.5 (76.1, 77.0) | 4.71 (4.17, 5.28) | 94.2 (94.0, 94.3) | 4.46 (3.66, 5.54) | 94.0 (90.6, 96.5) | 5.87 (3.52, 9.74) |
| | ≥6 | 82.4 (82.0, 82.7) | 5.17 (4.61, 5.82) | 94.1 (92.2, 95.5) | 5.22 (4.32, 6.32) | 93.0 (88.4, 95.8) | 6.43 (3.85, 10.83) |
| Urgent care (or higher) | | | | | | | |
| | 0 | 38.2 (37.8, 38.6) | 3.51 (3.22, 3.83) | 75.0 (74.0, 75.5) | 3.25 (2.84, 3.70) | 75.6 (73.6, 77.6) | 4.19 (2.97, 5.90) |
| | 1-2 | 50.8 (50.2, 51.4) | 4.06 (3.58, 4.59) | 78.6 (78.1, 79.1) | 3.77 (3.14, 4.51) | 84.0 (80.5, 86.7) | 5.04 (3.07, 8.20) |
| | 3-5 | 68.3 (67.7, 68.8) | 4.69 (4.20, 5.33) | 87.3 (87.0, 87.6) | 4.54 (3.72, 5.57) | 90.8 (86.7, 93.6) | 6.26 (3.91, 10.22) |
| | ≥6 | 78.0 (77.5, 78.4) | 5.39 (4.78, 6.13) | 88.3 (85.8, 90.5) | 5.35 (4.47, 6.47) | 90.0 (85.2, 93.2) | 6.63 (4.09, 10.88) |
| Emergency department (*or higher*) | | | | | | | |
| | 0 | 16.2 (16.0, 16.5) | 3.73 (3.43, 4.06) | 32.0 (31.6, 32.5) | 3.52 (3.10, 4.00) | 63.8 (61.4, 66.0) | 4.30 (3.10, 5.83) |
| | 1-2 | 28.3 (27.8, 28.8) | 4.44 (3.98, 5.01) | 41.2 (40.4, 42.0) | 4.17 (3.48, 4.96) | 78.3 (74.4, 81.7) | 5.30 (3.26, 7.95) |
| | 3-5 | 52.2 (51.5, 52.8) | 5.06 (4.48, 5.69) | 66.0 (65.3, 66.7) | 5.04 (4.19, 5.95) | 88.4 (84.3, 91.7) | 6.51 (4.16, 10.23) |
| | ≥6 | 68.8 (68.2, 69.3) | 5.53 (4.97, 6.18) | 73.8 (73.2, 74.4) | 5.77 (4.84, 6.92) | 88.5 (83.4, 91.9) | 6.77 (4.25, 10.66) |
| Inpatient admission (*or higher*) | | | | | | | |
| | 0 | 1.8 (1.8, 1.9) | 5.95 (5.43, 6.58) | 1.8 (1.6, 2.0) | 5.79 (4.87, 6.87) | 15.0 (13.0, 17.4) | 5.35 (3.98, 7.19) |
| | 1-2 | 6.5 (6.3, 6.7) | 6.39 (5.64, 7.33) | 6.7 (6.4, 6.8) | 6.03 (4.82, 7.72) | 37.2 (33.3, 41.0) | 6.24 (4.17, 9.73) |
| | 3-5 | 21.4 (20.7, 21.9) | 6.95 (6.06, 7.94) | 23.0 (22.3, 23.6) | 7.06 (5.47, 9.09) | 59.0 (54.0, 63.9) | 6.66 (4.32, 10.22) |
| | ≥6 | 38.1 (37.4, 39.0) | 7.50 (6.54, 8.58) | 35.1 (33.9, 36.1) | 7.68 (6.09, 9.80) | 69.0 (63.1, 74.3) | 7.14 (4.78, 10.81) |
| Mechanical ventilation (*or higher*) | | | | | | | |
| | 0 | 0.3 (0.2, 0.3) | 14.14 (12.15, 16.53) | 0.2 (0.1, 0.3) | 12.41 (8.39, 18.07) | 0.9 (0.4, 1.8) | 15.62 (8.68, 27.98) |
| | 1-2 | 1.1 (1.0, 1.1) | 16.49 (13.17, 20.64) | 0.8 (0.7, 1.1) | 13.29 (7.76, 21.79) | 4.0 (2.5, 6.6) | 11.78 (5.08, 25.94) |
| | 3-5 | 4.3 (4.2, 4.4) | 16.60 (13.53, 20.53) | 3.4 (2.6, 4.3) | 18.60 (10.91, 30.19) | 9.2 (6.2, 13.5) | 11.58 (5.04, 25.63) |
| | ≥6 | 10.3 (10.1, 10.7) | 17.49 (13.99, 21.93) | 7.4 (6.1, 9.1) | 15.60 (8.69, 26.91) | 11.5 (7.8, 17.1) | 16.06 (7.07, 30.63) |
| Death | | | | | | | |
| | 0 | 0.2 (0.2, 0.3) | 23.43 (19.89, 27.58) | 0.1 (0.0, 0.1) | 22.84 (14.95, 34.49) | 0.1 (0.0, 0.8) | 32.04 (15.26, 63.21) |
| | 1-2 | 0.8 (0.8, 0.9) | 24.62 (19.54, 31.27) | 0.5 (0.3, 0.6) | 20.37 (11.59, 36.87) | 1.6 (0.7, 3.3) | 22.06 (7.86, 59.92) |
| | 3-5 | 3.5 (3.4, 3.6) | 24.70 (19.62, 30.87) | 2.4 (1.9, 3.1) | 25.89 (14.34, 47.07) | 4.8 (2.8, 8.6) | 25.41 (8.91, 62.13) |
| | ≥6 | 9.0 (8.7, 9.3) | 25.14 (19.81, 31.69) | 5.8 (4.6, 7.1) | 21.50 (11.62, 38.69) | 7.5 (4.4, 12.4) | 22.45 (8.37, 54.55) |

We report estimates from best-fitting distributions, based on models yielding the minimum AIC score.
