# Supplementary material for: Clinical progression parameters associated with SARS-CoV-2, influenza, and respiratory syncytial virus infections in a large US integrated healthcare population
Source: PLoS Comput Biol. 2025 Nov 19;21(11):e1013723. doi: 10.1371/journal.pcbi.1013723 (PMC12643285; doi:10.1371/journal.pcbi.1013723)
Supplement: S1 File — (ZIP) [file pcbi.1013723.s001.zip › S1 File/S11_Table.pdf]

S11 Table: Proportions of cases attaining or exceeding each acuity threshold, by sex.

Acuity threshold	Stratum	SARS-CoV-2 infections		Influenza infections		RSV infections	
		Proportion, % (95% CI)	*Median time from symptoms onset to event, days (95% CI)*	*Proportion, % (95% CI)*	*Median time from symptoms onset to event, days (95% CI)*	*Proportion, % (95% CI)*	*Median time from symptoms onset to event, days (95% CI)*
Virtual care (or higher)							
	Male	70.1 (69.7, 70.4)	3.75 (3.25, 4.39)	92.9 (92.7, 93.0)	3.30 (2.75, 3.99)	92.8 (90.9, 94.4)	4.51 (4.02, 5.11)
	Female	69.9 (69.4, 70.5)	4.00 (3.62, 4.42)	93.4 (93.1, 93.6)	3.46 (3.02, 4.01)	92.2 (89.8, 93.8)	4.85 (4.49, 5.27)
Outpatient office visit (or higher)							
	Male	57.7 (57.0, 58.3)	3.98 (3.45, 4.63)	89.1 (88.8, 89.4)	3.41 (2.78, 4.19)	90.5 (88.3, 92.1)	4.67 (4.15, 5.23)
	Female	55.2 (54.8, 55.7)	4.3 (3.87, 4.79)	86.5 (86.2, 86.8)	3.60 (3.14, 4.12)	90.0 (87.7, 91.9)	4.89 (4.49, 5.32)
Urgent care (or higher)							
	Male	50.7 (50.1, 51.3)	3.91 (3.37, 4.58)	79.6 (79.1, 80.1)	3.44 (2.89, 4.14)	83.1 (80.8, 85.2)	4.74 (4.25, 5.25)
	Female	46.8 (46.4, 47.2)	4.13 (3.73, 4.59)	75.6 (75.2, 76.0)	3.65 (3.22, 4.11)	80.1 (78.4, 81.6)	5.23 (4.86, 5.61)
Emergency department (*or higher*)							
	Male	30.1 (29.6, 30.6)	4.59 (4.09, 5.14)	38.5 (37.8, 39.2)	3.90 (3.22, 4.63)	75.4 (74.9, 75.9)	4.90 (4.46, 5.42)
	Female	26.0 (26.0, 26.7)	4.36 (3.77, 5.05)	38.5 (37.9, 39.0)	4.08 (3.59, 4.65)	72.5 (71.8, 73.3)	5.52 (5.17, 5.94)
Inpatient admission (*or higher*)							
	Male	9.9 (9.6, 10.2)	6.77 (5.6, 8.16)	5.9 (5.8, 6.1)	6.85 (5.01, 9.55)	36.7 (34.3, 39.3)	6.09 (5.51, 6.75)
	Female	6.7 (6.5, 6.8)	6.94 (6.06, 7.93)	5.8 (5.7, 5.9)	6.37 (5.05, 7.92)	30.3 (28.5, 32.3)	6.55 (6.1, 7.05)
Mechanical ventilation (*or higher*)							
	Male	2.3 (2.2, 2.3)	17.10 (12.93, 23.77)	1.0 (0.9, 1.1)	13.45 (11.66, 15.60)	4.9 (3.6, 6.4)	16.47 (12.85, 21.25)
	Female	1.3 (1.2, 1.3)	16.34 (12.93, 20.31)	0.8 (0.6, 0.9)	12.72 (11.51, 14.07)	3.5 (2.4, 5.1)	11.79 (9.83, 14.24)
Death							
	Male	2.1 (2.0, 2.1)	25.15 (17.94, 36.05)	0.6 (0.5, 0.7)	24.75 (20.29, 29.42)	2.4 (1.6, 3.6)	22.53 (20.92, 24.23)
	Female	1.1 (1.0, 1.1)	24.33 (18.77, 31.16)	0.5 (0.4, 0.7)	20.92 (18.25, 23.96)	1.8 (1.0, 3.0)	24.49 (23.18, 25.87)

We report estimates from best-fitting distributions, based on models yielding the minimum AIC score.
