# Supplementary material for: Clinical progression parameters associated with SARS-CoV-2, influenza, and respiratory syncytial virus infections in a large US integrated healthcare population
Source: PLoS Comput Biol. 2025 Nov 19;21(11):e1013723. doi: 10.1371/journal.pcbi.1013723 (PMC12643285; doi:10.1371/journal.pcbi.1013723)
Supplement: S1 File — (ZIP) [file pcbi.1013723.s001.zip › S1 File/S12_Table.pdf]

S12 Table: Proportions of cases attaining or exceeding each acuity threshold, by neighborhood deprivation index.

Acuity threshold	Stratum	SARS-CoV-2 infections		Influenza infections		RSV infections	
		Proportion, % (95% CI)	*Median time from symptoms onset to event, days (95% CI)*	*Proportion, % (95% CI)*	*Median time from symptoms onset to event, days (95% CI)*	*Proportion, % (95% CI)*	*Median time from symptoms onset to event, days (95% CI)*
Virtual care (or higher)							
	Below –1	70.0 (69.5, 70.5)	4.02 (3.78, 4.28)	91.0 (89.0, 92.7)	3.60 (3.30, 3.93)	88.2 (79.9, 93.6)	5.11 (3.86, 6.73)
	–1 ≤ NDI < 0	71.3 (69.5, 73.0)	4.00 (3.66, 4.38)	92.4 (92.1, 92.7)	3.44 (3.04, 3.92)	94.0 (91.5, 95.6)	4.92 (3.24, 7.40)
	0 ≤ NDI < 1	70.1 (69.6, 70.6)	3.84 (3.53, 4.17)	93.7 (93.5, 93.9)	3.40 (2.99, 3.84)	93.7 (91.4, 95.4)	4.33 (3.01, 6.32)
	Above 1	69.5 (68.9, 70.0)	3.78 (3.46, 4.13)	93.3 (93.0, 93.6)	3.24 (2.86, 3.66)	91.4 (87.1, 94.2)	4.85 (3.30, 7.01)
Outpatient office visit (or higher)							
	Below –1	55.6 (54.9, 56.2)	4.25 (4, 4.51)	84.9 (82.4, 87.2)	3.82 (3.51, 4.17)	82.9 (73.4, 89.7)	5.21 (4.06, 6.68)
	–1 ≤ NDI < 0	59.0 (58.6, 59.4)	4.32 (3.93, 4.7)	86.9 (86.5, 87.3)	3.56 (3.15, 4.03)	91.4 (89.3, 93.3)	5.00 (3.50, 7.18)
	0 ≤ NDI < 1	56.6 (56.1, 57.2)	4.1 (3.72, 4.49)	88.2 (87.8, 88.6)	3.53 (3.14, 3.96)	92.3 (90.2, 93.9)	4.48 (3.07, 6.53)
	Above 1	55.3 (54.7, 55.9)	3.99 (3.64, 4.36)	88.6 (88.2, 89.0)	3.33 (2.96, 3.77)	89.1 (85.0, 92.1)	4.89 (3.39, 7.11)
Urgent care (or higher)							
	Below –1	47.4 (46.8, 48.0)	4.14 (3.89, 4.38)	72.0 (68.8, 74.7)	3.88 (3.61, 4.17)	75.0 (63.7, 83.4)	5.22 (4.17, 6.73)
	–1 ≤ NDI < 0	49.9 (49.4, 50.3)	4.18 (3.82, 4.57)	75.6 (75.0, 76.2)	3.59 (3.22, 4.00)	82.3 (79.6, 84.7)	5.13 (3.76, 7.14)
	0 ≤ NDI < 1	47.8 (47.2, 48.5)	4.01 (3.67, 4.39)	78.3 (77.7, 78.8)	3.55 (3.20, 3.96)	83.6 (81.1, 85.8)	4.68 (3.44, 6.59)
	Above 1	47.5 (46.8, 48.1)	3.82 (3.48, 4.16)	79.3 (78.8, 79.9)	3.43 (3.08, 3.81)	82.0 (77.6, 85.8)	5.12 (3.68, 7.36)
Emergency department (*or higher*)							
	Below –1	27.5 (27.0, 28.1)	4.55 (4.26, 4.88)	35.4 (32.2, 38.8)	4.35 (4.00, 4.69)	69.7 (58.0, 78.5)	5.64 (4.64, 6.98)
	–1 ≤ NDI < 0	33.2 (32.8, 33.6)	4.71 (4.34, 5.22)	36.1 (35.3, 36.8)	3.98 (3.55, 4.42)	73.7 (70.5, 76.5)	5.42 (3.96, 7.34)
	0 ≤ NDI < 1	28.3 (27.8, 28.8)	4.47 (4.06, 4.91)	39.3 (38.6, 40.0)	4.12 (3.70, 4.59)	75.9 (73.2, 78.5)	4.88 (3.60, 6.71)
	Above 1	26.5 (26.0, 27.0)	4.28 (3.90, 4.70)	39.4 (38.6, 40.2)	3.89 (3.49, 4.33)	74.5 (69.0, 79.1)	5.28 (3.86, 7.22)
Inpatient admission (*or higher*)							
	Below –1	8.5 (8.2, 8.7)	6.92 (6.39, 7.5)	5.3 (4.0, 6.9)	7.59 (6.69, 8.64)	35.5 (25.5, 47.4)	6.46 (5.15, 8.16)
	–1 ≤ NDI < 0	9.7 (8.6, 11.0)	6.68 (5.94, 7.44)	6.1 (5.9, 6.2)	6.29 (5.20, 7.72)	31.2 (28.2, 34.4)	6.75 (4.91, 9.29)
	0 ≤ NDI < 1	8.8 (8.5, 9.0)	6.96 (6.24, 7.79)	6.2 (6.0, 6.3)	6.76 (5.64, 8.16)	36.0 (33.2, 38.9)	6.01 (4.39, 8.23)
	Above 1	7.7 (7.4, 7.9)	7.02 (6.29, 7.88)	5.4 (5.2, 5.5)	6.77 (5.64, 8.08)	34.1 (29.1, 39.4)	5.94 (4.31, 8.19)
Mechanical ventilation (*or higher*)							
	Below –1	1.8 (1.7, 1.8)	14.63 (12.59, 16.92)	0.5 (0.2, 1.3)	24.40 (17.94, 33.21)	3.9 (1.2, 12.2)	17.86 (10.28, 31.21)
	–1 ≤ NDI < 0	2.2 (1.7, 2.8)	17.41 (14.05, 21.27)	0.8 (0.6, 1.0)	13.30 (8.59, 20.35)	4.1 (2.8, 6.3)	14.86 (7.27, 30.00)
	0 ≤ NDI < 1	1.7 (1.6, 1.7)	16.18 (13.13, 20.04)	1.0 (0.8, 1.2)	11.91 (7.66, 18.37)	4.1 (2.7, 6.2)	13.84 (6.72, 29.12)
	Above 1	1.7 (1.6, 1.7)	16.96 (13.81, 21.01)	0.9 (0.7, 1.2)	14.54 (9.18, 22.65)	4.5 (2.5, 7.7)	10.20 (4.81, 21.43)
Death							
	Below –1	1.5 (1.5, 1.6)	20.73 (17.49, 24.50)	0.3 (0.1, 1.1)	23.63 (16.16, 34.79)	2.6 (0.7, 10.7)	25.20 (11.69, 50.61)
	–1 ≤ NDI < 0	2.0 (1.5, 2.8)	25.92 (20.50, 32.27)	0.6 (0.5, 0.8)	23.03 (13.52, 38.35)	2.7 (1.6, 4.6)	20.13 (7.10, 52.93)
	0 ≤ NDI < 1	1.5 (1.5, 1.6)	24.01 (19.30, 30.12)	0.6 (0.4, 0.7)	22.15 (12.90, 38.04)	2.0 (1.1, 3.5)	27.01 (9.31, 73.35)
	Above 1	1.5 (1.4, 1.5)	26.86 (21.39, 33.29)	0.5 (0.3, 0.8)	23.18 (13.72, 39.69)	1.1 (0.4, 3.3)	28.15 (10.09, 77.42)

We report estimates from best-fitting distributions, based on models yielding the minimum AIC score.
