# Supplementary material for: Clinical progression parameters associated with SARS-CoV-2, influenza, and respiratory syncytial virus infections in a large US integrated healthcare population
Source: PLoS Comput Biol. 2025 Nov 19;21(11):e1013723. doi: 10.1371/journal.pcbi.1013723 (PMC12643285; doi:10.1371/journal.pcbi.1013723)
Supplement: S1 File — (ZIP) [file pcbi.1013723.s001.zip › S1 File/S13_Table.pdf]

S13 Table: Proportions of cases attaining or exceeding each acuity threshold, by vaccination status.

Acuity threshold	Stratum	SARS-CoV-2 infections		Influenza infections		RSV infections	
		Proportion, % (95% CI)	*Median time from symptoms onset to event, days (95% CI)*	*Proportion, % (95% CI)*	*Median time from symptoms onset to event, days (95% CI)*	*Proportion, % (95% CI)*	*Median time from symptoms onset to event, days (95% CI)*
Virtual care (or higher)							
	Unvaccinated	72.8 (72.3, 73.3)	3.66 (3.22, 4.10)	92.6 (92.4, 92.8)	3.19 (2.80, 3.65)	92.4 (91.1, 93.7)	4.69 (3.39, 6.59)
	Vaccinated[1]	68.4 (68.0, 68.7)	3.70 (3.39, 4.05)	93.5 (93.2, 93.7)	3.55 (2.95, 4.25)	96.3 (76.5, 99.5)	4.65 (2.88, 7.31)
	COVID-19: ≥3 doses	70.0 (69.5, 70.5)	4.00 (3.53, 4.51)	– –	– –	– –	– –
Outpatient office visit (or higher)							
	Unvaccinated	61.3 (60.7, 61.9)	3.83 (3.42, 4.31)	86.8 (86.5, 87.1)	3.32 (2.90, 3.75)	90.2 (88.6, 91.5)	4.79 (3.46, 6.58)
	Vaccinated[1]	55.8 (55.4, 56.2)	3.87 (3.55, 4.21)	88.4 (88.1, 88.8)	3.68 (3.02, 4.41)	96.3 (79.0, 99.5)	4.65 (2.90, 7.71)
	COVID-19: ≥3 doses	55.6 (54.9, 56.2)	4.31 (3.86, 4.87)	– –	– –	– –	– –
Urgent care (or higher)							
	Unvaccinated	53.6 (53.0, 54.2)	3.73 (3.32, 4.23)	75.9 (75.6, 76.4)	3.35 (2.97, 3.73)	81.6 (81.4, 81.8)	5.00 (3.82, 6.35)
	Vaccinated[1]	48.5 (48.0, 48.9)	3.77 (3.45, 4.14)	78.6 (78.0, 79.0)	3.72 (3.13, 4.42)	88.9 (72.5, 96.2)	5.13 (3.52, 7.20)
	COVID-19: ≥3 doses	47.4 (46.8, 48.0)	4.17 (3.66, 4.77)	– –	– –	– –	– –
Emergency department (*or higher*)							
	Unvaccinated	33.3 (32.7, 33.8)	3.98 (3.53, 4.46)	28.9 (28.5, 29.4)	3.87 (3.47, 4.34)	74.0 (73.5, 74.5)	5.22 (4.21, 6.43)
	Vaccinated[1]	25.8 (25.4, 26.1)	4.19 (3.85, 4.55)	46.3 (45.6, 47.1)	4.07 (3.46, 4.76)	77.9 (59.9, 89.5)	5.78 (4.33, 7.75)
	COVID-19: ≥3 doses	27.5 (27.0, 28.1)	4.67 (4.16, 5.26)	– –	– –	– –	– –
Inpatient admission (*or higher*)							
	Unvaccinated	7.7 (7.4, 7.9)	6.44 (5.68, 7.31)	3.1 (2.8, 3.5)	6.44 (5.29, 7.77)	33.7 (33.3, 34.0)	6.35 (5.23, 7.67)
	Vaccinated[1]	6.2 (6.1, 6.4)	6.1 (5.59, 6.71)	8.1 (7.9, 8.3)	6.64 (5.07, 8.60)	40.7 (24.5, 59.3)	6.90 (5.32, 9.11)
	COVID-19: ≥3 doses	8.5 (8.2, 8.7)	7.07 (6.25, 8.04)	– –	– –	– –	– –
Mechanical ventilation (*or higher*)							
	Unvaccinated	1.4 (1.3, 1.5)	16.17 (13.81, 18.82)	0.4 (0.3, 0.6)	12.74 (11.63, 13.99)	4.3 (3.3, 5.3)	13.21 (8.61, 20.48)
	Vaccinated[1]	1.3 (1.2, 1.5)	15.62 (12.49, 19.41)	1.2 (1.1, 1.3)	16.12 (14.07, 18.43)	3.7 (0.5, 19.2)	33.81 (18.68, 62.50)
	COVID-19: ≥3 doses	1.8 (1.7, 1.8)	17.02 (13.58, 21.03)	– –	– –	– –	– –
Death							
	Unvaccinated	1.0 (1.0, 1.9)	23.01 (17.83, 29.09)	0.3 (0.2, 0.4)	20.29 (18.11, 22.71)	2.2 (1.5, 3.0)	– –
	Vaccinated[1]	1.2 (1.0, 1.4)	24.54 (20.71, 29.15)	0.8 (0.7, 0.8)	23.37 (19.96, 27.26)	– –	– –
	COVID-19: ≥3 doses	1.5 (1.5, 1.6)	25.07 (19.45, 32.17)	– –	– –	– –	– –

We report estimates from best-fitting distributions, based on models yielding the minimum AIC score. Missing time-to-event estimates indicate either a lack of model convergence or a confidence interval wider than the follow-up period due to sparse observations.

[1]For SARS-CoV-2 infections, rows correspond to a category of "under-vaccinated" individuals who received 1-2 total COVID-19 vaccine doses. For influenza infections, individuals are considered vaccinated if they received seasonal influenza vaccine for the applicable season; for RSV infections, individuals are considered vaccinated if they received any RSV vaccine.
