# Supplementary material for: Clinical progression parameters associated with SARS-CoV-2, influenza, and respiratory syncytial virus infections in a large US integrated healthcare population
Source: PLoS Comput Biol. 2025 Nov 19;21(11):e1013723. doi: 10.1371/journal.pcbi.1013723 (PMC12643285; doi:10.1371/journal.pcbi.1013723)
Supplement: S1 File — (ZIP) [file pcbi.1013723.s001.zip › S1 File/S14_Table.pdf]

S14 Table: Stratified hospital length of stay estimates for admissions associated with each viral infection and leading to discharge or mortality.

Outcome	Stratum	SARS-CoV-2 infections		Influenza infections		RSV infections	
		Proportion, % (95% CI)	*Median time from symptoms onset to event, days (95% CI)*	*Proportion, % (95% CI)*	*Median time from symptoms onset to event, days (95% CI)*	*Proportion, % (95% CI)*	*Median time from symptoms onset to event, days (95% CI)*
Any							
	Age						
	0-17y	– –	3.91 (3.67, 4.20)	– –	3.65 (3.28, 4.08)	– –	3.30 (2.92, 3.73)
	18-49y	– –	3.46 (2.94, 4.06)	– –	3.67 (3.08, 4.33)	– –	4.18 (3.70, 4.73)
	50-59y	– –	4.23 (3.59, 4.96)	– –	3.90 (3.28, 4.60)	– –	5.83 (5.09, 6.63)
	60-69y	– –	4.17 (3.55, 4.90)	– –	4.20 (3.53, 4.96)	– –	4.55 (3.97, 5.17)
	70-79y	– –	4.49 (3.82, 5.28)	– –	4.10 (3.44, 4.84)	– –	4.54 (3.96, 5.16)
	80-89y	– –	4.30 (3.65, 5.05)	– –	4.39 (3.69, 5.18)	– –	4.56 (3.98, 5.19)
	≥90y	– –	4.28 (3.63, 5.03)	– –	3.83 (3.22, 4.52)	– –	4.70 (4.10, 5.35)
	Sex	– –		– –		– –	
	Male	– –	4.36 (4.23, 4.48)	– –	4.07 (3.84, 4.32)	– –	4.07 (3.76, 3.84)
	Female	– –	4.12 (4.00, 4.23)	– –	3.86 (3.64, 4.09)	– –	4.52 (4.19, 4.88)
	Race/ethnicity	– –		– –		– –	
	White, non-Hispanic	– –	4.22 (3.98, 4.49)	– –	4.04 (3.56, 4.58)	– –	4.26 (3.56, 5.13)
	Asian, non-Hispanic	– –	3.99 (3.76, 4.24)	– –	3.90 (3.44, 4.43)	– –	4.38 (3.67, 5.28)
	Black, non-Hispanic	– –	4.74 (4.47, 5.04)	– –	4.25 (3.75, 4.83)	– –	4.92 (4.11, 5.93)
	Hispanic (any race)	– –	4.13 (3.89, 4.39)	– –	3.81 (3.36, 4.33)	– –	4.25 (3.55, 5.12)
	Pacific Islander	– –	4.42 (4.16, 4.69)	– –	4.47 (4.29, 5.53)	– –	5.65 (4.72, 6.81)
	Native American/Alaska native	– –	4.84 (4.56, 5.14)	– –	5.96 (5.25, 6.76)	– –	3.30 (2.76, 3.98)
	Other	– –	4.56 (2.61, 7.60)	– –	3.42 (3.01, 3.88)	– –	6.32 (5.29, 7.62)
	Multiple	– –	5.02 (3.42, 7.34)	– –	1.00 (0.88, 1.14)	– –	2.00 (1.67, 2.41)
	Unknown	– –	3.73 (3.51, 3.96)	– –	1.41 (1.25, 1.61)	– –	2.00 (1.67, 2.41)
	NDI	– –		– –		– –	
	Below –1	– –	4.09 (3.74, 4.48)	– –	3.69 (3.42, 3.97)	– –	4.02 (3.59, 4.51)
	–1 ≤ NDI < 0	– –	4.21 (3.84, 4.60)	– –	3.75 (3.02, 4.74)	– –	4.08 (3.06, 5.44)
	0 ≤ NDI < 1	– –	4.23 (3.86, 4.62)	– –	4.01 (3.23, 5.07)	– –	4.68 (3.52, 6.24)
	Above 1	– –	4.43 (4.04, 4.84)	– –	4.02 (3.23, 5.07)	– –	4.49 (3.37, 5.98)
	Charlson comorbidity index	– –		– –		– –	
	0	– –	3.58 (3.38, 3.78)	– –	3.90 (3.52, 4.30)	– –	3.94 (3.48, 4.44)
	1-2	– –	4.09 (3.86, 4.33)	– –	3.58 (3.24, 3.95)	– –	4.03 (3.50, 4.65)
	3-5	– –	4.33 (4.09, 4.58)	– –	3.95 (3.57, 4.36)	– –	4.72 (4.10, 5.46)
	≥6	– –	4.47 (4.22, 4.73)	– –	4.50 (4.06, 4.96)	– –	4.47 (3.89, 5.17)
	Unvaccinated	– –	4.38 (4.16, 4.61)	– –	3.93 (3.57, 4.32)	– –	4.33 (4.07, 4.59)
	Vaccinated[1]	– –	4.39 (4.17, 4.62)	– –	3.96 (3.61, 4.36)	– –	4.38 (4.11, 4.64)
	COVID-19: ≥3 doses	– –	4.18 (3.97, 4.40)	– –	– –	– –	– –
Discharged alive							
	Age						
	0-17y	100 (0, 100)	3.91 (3.67, 4.20)	100 (0, 100)	3.65 (3.28, 4.08)	100 (0, 100)	3.30 (2.92, 3.73)
	18-49y	97.9 (96.2, 98.8)	5.37 (4.93, 5.85)	96.9 (93.4, 98.5)	3.74 (3.24, 4.36)	100 (0, 100)	4.18 (3.70, 4.73)
	50-59y	95.5 (92.9, 97.2)	5.14 (4.85, 5.44)	98.2 (93.1, 99.5)	4.23 (3.80, 4.71)	95.9 (50.8, 99.8)	5.74 (5.08, 6.49)
	60-69y	94 (92.1, 95.5)	5.22 (5.01, 5.44)	94.6 (90.7, 97)	4.04 (3.73, 4.39)	98.9 (75.9, 1.00)	4.63 (4.10, 5.23)
	70-79y	93.1 (91.7, 94.2)	4.72 (4.53, 4.92)	96.7 (94.3, 98.1)	4.34 (3.91, 4.82)	97.4 (63.3, 99.9)	4.38 (3.87, 4.95)
	80-89y	91.5 (90, 92.8)	4.43 (4.15, 4.73)	92.8 (88.5, 95.4)	3.82 (3.14, 4.69)	98.7 (72.0, 99.9)	4.52 (4.00, 5.11)
	≥90y	89 (86.1, 91.4)	3.91 (3.67, 4.20)	94.2 (84.6, 97.8)	3.65 (3.28, 4.08)	96.1 (51.9, 99.8)	4.51 (4.00, 5.10)
	Sex						

	Male	92.3 (91.3, 93.2)	5.01 (4.85, 5.16)	95.6 (93.6, 97.0)	4.01 (3.78, 4.29)	98 (95.4, 99.2)	4.03 (3.66, 4.43)
	Female	93.8 (92.9, 94.7)	4.71 (4.57, 4.87)	96.1 (94.2, 97.3)	3.82 (3.61, 4.08)	98.6 (96.8, 99.4)	4.46 (4.12, 4.84)
	Race/ethnicity						
	White, non-Hispanic	92.7 (91.5, 93.6)	5.51 (5.18, 5.85)	95.0 (93.8, 96.0)	5.18 (4.54, 5.89)	98.7 (76.3, 99.9)	4.18 (3.48, 4.96)
	Asian, non-Hispanic			95.3 (93.0, 96.9)	3.83 (3.35, 4.35)	98.6 (86.9, 99.8)	4.35 (3.62, 5.15)
		92.9 (90.5, 94.8)	4.69 (4.38, 5.01)				
	Black, non-Hispanic	93.3 (91.2, 95.1)	5.51 (5.18, 5.85)	97.4 (96.7, 97.9)	4.3 (3.76, 4.89)		
	Hispanic (any race)	93.6 (92.4, 94.7)	4.82 (4.64, 5.01)	96.4 (95.5, 97.2)	3.74 (3.27, 4.25)	97.4 (62.0, 99.9)	4.18 (3.47, 4.95)
	Pacific Islander	86 (72.6, 93.6)	4.73 (4.57, 4.89)	– –	– –	– –	– –
	Native American/Alaska native	94.1 (66.6, 99.2)	6.23 (4.87, 7.96)	– –	– –	– –	– –
	Other	100 (0, 100)	4.56 (2.61, 7.60)	– –	– –	– –	– –
	Multiple	100 (0, 100)	5.02 (3.42, 7.34)	– –	– –	– –	– –
	Unknown	88.9 (52.3, 98.5)	4.69 (4.38, 5.01)	– –	– –	– –	– –
	NDI						
	Below –1	92.2 (88.5, 95)	4.41 (3.99, 4.83)	100 (0, 100)	3.69 (3.42, 3.97)	100 (0, 100)	4.02 (3.59, 4.51)
	–1 ≤ NDI < 0	93.3 (92.1, 94.4)	4.79 (4.61, 4.97)	95.6 (93.0, 97.3)	3.93 (3.67, 4.23)	98.4 (94.6, 99.4)	4.60 (4.16, 5.11)
	0 ≤ NDI < 1	92.5 (91.1, 93.6)	4.94 (4.75, 5.14)	95 (92.5, 96.7)	4.04 (3.65, 4.45)	97.3 (94, 98.8)	4.47 (3.84, 5.13)
	Above 1	93.4 (91.6, 94.8)	5 (4.73, 5.26)	96.6 (93.3, 98.3)	3.69 (3.42, 3.97)	99 (93.3, 99.8)	4.02 (3.59, 4.51)
	Charlson comorbidity index						
	0	96.1 (94.4, 97.3)	4.35 (4.09, 4.62)	98.3 (95.3, 99.3)	3.81 (3.42, 4.23)	100 (0, 100)	3.94 (3.48, 4.44)
	1-2	94.4 (93.1, 95.6)	4.79 (4.57, 5.01)	95.9 (93.2, 97.5)	3.61 (3.33, 3.92)	98 (94.2, 99.4)	4.65 (4.15, 5.15)
	3-5	93.1 (91.8, 94.2)	4.89 (4.71, 5.08)	97 (94.7, 98.4)	3.89 (3.59, 4.23)	98.4 (95.2, 99.4)	4.44 (3.93, 4.95)
	≥6	91 (89.6, 92.4)	5.09 (4.9, 5.29)	92.8 (89.6, 95.1)	4.43 (4.06, 4.86)	97.5 (93.8, 99.1)	3.94 (3.48, 4.44)
	Unvaccinated	93.1 (90.8, 94.9)	5.24 (4.93, 5.56)	95.7 (92.7, 97.5)	3.85 (3.49, 4.22)	98.3 (97, 99.1)	4.28 (4.09, 4.53)
	Vaccinated[1]	93.7 (91.8, 95.3)	5.07 (4.78, 5.36)	96 (94.8, 97.2)	3.93 (3.73, 4.13)	100 (0, 100)	4.38 (4.11, 4.64)
	COVID-19: ≥3 doses	92.9 (92.1, 93.7)	4.76 (4.64, 4.89)	– –	– –	– –	– –
In-hospital mortality							
	Age						
	0-17y	– –	– –	– –	– –	– –	– –
	18-49y	2.1 (1.2, 3.8)	7.93 (5.31, 11.61)	3.1 (1.5, 6.6)	28.97 (9.05, 87.46)	– –	– –
	50-59y	4.5 (2.8, 7.1)	8.21 (6.32, 10.37)	1.8 (0.5, 6.9)	4.4 (2.7, 7.3)	4.1 (0.0, 50.2)	– –
	60-69y	6 (4.5, 7.9)	8.97 (7.59, 10.49)	5.4 (3, 9.3)	7.03 (4.18, 11.85)	1.1 (0.0, 24.1)	– –
	70-79y	6.9 (5.8, 8.3)	7.07 (6.03, 8.36)	3.3 (1.9, 5.7)	7.59 (4.9, 11.89)	2.6 (0.0, 36.7)	– –
	80-89y	8.5 (7.2, 10)	6.34 (4.99, 7.86)	7.2 (4.6, 11.5)	3.35 (1.4, 8.17)	1.3 (0.0, 28.0)	– –
	≥90y	11 (8.6, 13.9)	9.29 (5.29, 15.94)	5.8 (2.2, 15.4)	4.56 (2.24, 8.93)	4.0 (0.0, 48.1)	– –
	Male	7.7 (6.8, 8.7)	8.45 (7.51, 9.57)	4.4 (3.0, 6.4)	4.87 (3.25, 7.3)	2 (0.8, 4.6)	8.13 (3.76, 13.63)
	Female	6.2 (5.3, 7.1)	6.83 (5.97, 7.82)	3.9 (2.7, 5.8)	5.66 (3.73, 8.58)	1.4 (0.6, 3.2)	16.15 (6.47, 22.96)
	Race/ethnicity						
	White, non-Hispanic	7.3 (6.4, 8.5)	9.39 (7.41, 11.82)	4.9 (4.0, 6.2)	4.85 (2.36, 10.17)	7.3 (6.4, 8.5)	9.39 (7.41, 11.82)
	Asian, non-Hispanic	7.1 (5.2, 9.5)	7.25 (5.6, 9.68)	4.7 (3.1, 7.0)	5.67 (2.76, 11.89)	1.4 (0.0, 13.1)	7.00 (1.83, 43.59)
	Black, non-Hispanic	6.7 (4.9, 8.8)	9.39 (7.41, 11.82)	2.6 (2.0, 3.3)	2.91 (1.42, 6.11)	– –	– –
	Hispanic (any race)	6.4 (5.3, 7.6)	8.95 (7.67, 10.58)	3.6 (2.8, 4.5)	6.52 (3.17, 13.67)	2.6 (0.0, 38.0)	7.73 (3.22, 81.22)
	Pacific Islander	14 (6.4, 27.4)	6.76 (5.9, 7.75)	– –	– –	– –	– –
	Native American/Alaska native	5.9 (0.8, 33.4)	7.2 (3.65, 13.91)	– –	– –	– –	– –
	Other	– –	– –	– –	– –	– –	– –
	Multiple	– –	– –	– –	– –	– –	– –
	Unknown	11.1 (1.5, 47.7)	7.05 (1.34, 34.36)	– –	– –	– –	– –
	NDI						
	Below –1	7.8 (5, 11.5)	7.25 (5, 10.04)	– –	– –	– –	9.78 (3.78, 18.13)

–1 ≤ NDI < 0	6.7 (5.6, 7.9)	7.64 (6.53, 8.95)	4.4 (2.7, 7.0)	6.14 (4, 9.31)	1.6 (0.6, 5.4)	14.29 (5.64, 20.86)
0 ≤ NDI < 1	7.5 (6.4, 8.9)	8.04 (7.02, 9.29)	5 (3.3, 7.5)	3.47 (1.71, 7.14)	2.7 (1.2, 6)	5.43 (0.92, 19.58)
Above 1	6.6 (5.2, 8.4)	8.17 (6.54, 10.11)	3.4 (1.7, 6.7)	5.48 (3.33, 9.18)	1 (0.2, 6.7)	9.78 (3.78, 18.13)
Charlson comorbidity index						
0	3.9 (2.7, 5.6)	7.78 (5.58, 11.05)	1.7 (0.7, 4.7)	14 (5.31, 34.63)	– –	– –
1-2	5.6 (4.4, 6.9)	7.48 (6.04, 9.15)	4.1 (2.5, 6.8)	3.07 (1.88, 4.95)	2 (0.6, 5.8)	12.68 (4.64, 23.4)
3-5	6.9 (5.8, 8.2)	8.06 (6.92, 9.36)	3 (1.6, 5.3)	6.49 (3.53, 11.29)	1.6 (0.6, 4.8)	11.42 (3.47, 21.16)
≥6	9 (7.6, 10.4)	7.51 (6.51, 8.64)	7.2 (4.9, 10.4)	5.51 (3.69, 8.09)	2.5 (0.9, 6.2)	10.28 (3.84, 22.14)
Unvaccinated	6.9 (5.1, 9.2)	8.17 (6.37, 10.68)	4.3 (2.5, 7.3)	6.51 (3.81, 11.16)	1.7 (0.9, 3)	– –
Vaccinated[1]	6.3 (4.7, 8.2)	5.79 (4.57, 7.23)	4 (2.8, 5.2)	4.89 (3.57, 6.79)	– –	– –
COVID-19: ≥3 doses	7.1 (6.3, 7.9)	8.01 (7.16, 8.96)	– –	– –	– –	– –

Missing time-to-event estimates indicate either a lack of model convergence or a confidence interval wider than the follow-up period due to sparse observations. Groups without a probability estimate had no events observed. Groups with no median time to event either had no events occur, or too few for model convergence.

[1]For SARS-CoV-2 infections, rows correspond to a category of “under-vaccinated” individuals who received 1-2 total COVID-19 vaccine doses. For influenza infections, individuals are considered vaccinated if they received seasonal influenza vaccine for the applicable season; for RSV infections, individuals are considered vaccinated if they received any RSV vaccine.
