# Supplementary material for: Clinical progression parameters associated with SARS-CoV-2, influenza, and respiratory syncytial virus infections in a large US integrated healthcare population
Source: PLoS Comput Biol. 2025 Nov 19;21(11):e1013723. doi: 10.1371/journal.pcbi.1013723 (PMC12643285; doi:10.1371/journal.pcbi.1013723)
Supplement: S1 File — (ZIP) [file pcbi.1013723.s001.zip › S1 File/S1_Table.pdf]

S1 Table: Acute respiratory illness diagnosis codes.

ICD-10-CM Code	Diagnosis
A48.1	Legionnaire's disease
B34.2	Coronavirus infection (unspecified)
B44.0	Invasive pulmonary aspergillosis
B97.29	Other coronavirus as the cause of diseases classified elsewhere
J00	Acute nasopharyngitis (common cold)
J01.00	Acute maxillary sinusitis, unspecified
J01.10	Acute frontal sinusitis, unspecified
J01.20	Acute ethmoidal sinusitis, unspecified
J01.30	Acute sphenoidal sinusitis, unspecified
J01.40	Acute pansinusitis, unspecified
J01.80	Other acute sinusitis
J01.90	Acute sinusitis, unspecified
J02.0	Streptococcal pharyngitis
J02.8	Acute pharyngitis due to other specified organisms
J02.9	Acute pharyngitis, unspecified
J03.00	Acute streptococcal tonsillitis, unspecified
J03.90	Acute tonsillitis, unspecified
J04.0	Acute laryngitis
J04.10	Acute tracheitis without obstruction
J05.0	Acute obstructive laryngitis (croup)
J05.10	Acute epiglottitis without obstruction
J06.0	Acute laryngopharyngitis
J06.9	Acute upper respiratory infection, unspecified
J09.X1	Influenza due to identified novel influenza A virus with pneumonia
J09.X2	Influenza due to identified novel influenza A virus with other respiratory manifestations
J10.00	Influenza due to other identified influenza virus with unspecified type of pneumonia
J10.01	Influenza due to other identified influenza virus with same other identified influenza virus pneumonia
J10.08	Influenza due to other identified influenza virus with other pneumonia
J10.1	Influenza due to other identified influenza virus with other respiratory manifestations
J10.2	Influenza due to other identified influenza virus with gastrointestinal manifestations
J11.00	Influenza due to unidentified influenza virus with unspecified type of pneumonia
J11.08	Influenza due to unidentified influenza virus with specified pneumonia
J11.1	Influenza due to unidentified influenza virus with other respiratory manifestations
J12.1	Respiratory syncytial virus pneumonia
J12.2	Parainfluenza virus pneumonia
J12.3	Human metapneumovirus pneumonia
J12.81	Pneumonia due to SARS-associated coronavirus
J12.82	Pneumonia due to coronavirus disease 2019
J12.89	Other viral pneumonia
J12.9	Viral pneumonia, unspecified
J13	Pneumonia due to *Streptococcus pneumoniae*
J14	Pneumonia due to *Haemophilus influenzae*
J15.0	Pneumonia due to *Klebsiella pneumoniae*
J15.1	Pneumonia due to *Pseudomonas*
J15.20	Pneumonia due to *Staphylococcus*, unspecified
J15.211	Pneumonia due to methicillin susceptible *Staphylococcus aureus*
J15.212	Pneumonia due to methicillin resistant *Staphylococcus aureus*
J15.4	Pneumonia due to other *Streptococci*
J15.5	Pneumonia due to *Escherichia coli*
J15.6	Pneumonia due to other aerobic gram-negative bacteria
J15.7	Pneumonia due to *Mycoplasma pneumoniae*
J15.8	Pneumonia due to other specified bacteria
J15.9	Unspecified bacterial pneumonia
J16.8	Pneumonia due to other specified infectious organisms
J18.0	Bronchopneumonia, unspecified organism
J18.1	Lobar pneumonia, unspecified organism
J18.8	Other pneumonia, unspecified organism
J18.9	Pneumonia, unspecified organism
J20.2	Acute bronchitis due to *Streptococcus*
J20.5	Acute bronchitis due to respiratory syncytial virus
J20.6	Acute bronchitis due to rhinovirus
J20.8	Acute bronchitis due to other specified organisms
J20.9	Acute bronchitis, unspecified
J22	Unspecified acute lower respiratory infection
J39.0	Retropharyngeal and parapharyngeal abscess
J39.1	Other abscess of pharynx
J39.2	Other diseases of pharynx
J39.8	Other specified diseases of upper respiratory tract
J80	Acute respiratory distress syndrome
J96.00	Acute respiratory failure, unspecified with hypoxia or hypercapnia
J96.01	Acute respiratory failure with hypoxia
J96.02	Acute respiratory failure with hypercapnia
J96.10	Chronic respiratory failure, unspecified with hypoxia or hypercapnia
J96.11	Chronic respiratory failure with hypoxia
J96.12	Chronic respiratory failure with hypercapnia

J96.20	Acute and chronic respiratory failure, unspecified with hypoxia or hypercapnia
J96.21	Acute and chronic respiratory failure with hypoxia
J96.22	Acute and chronic respiratory failure with hypercapnia
J96.90	Respiratory failure, unspecified with hypoxia or hypercapnia
J96.91	Respiratory failure with hypoxia
J96.92	Respiratory failure with hypercapnia
M35.81	Multisystem inflammatory syndrome
M35.89	Other specified systemic involvement of connective tissue
R05.1	Acute cough
R05.3	Chronic cough
R05.8	Other specified cough
R05.9	Cough, unspecified
R09.2	Respiratory arrest
R50.9	Fever, unspecified
U07.1	COVID-19
