# Supplementary material for: Clinical progression parameters associated with SARS-CoV-2, influenza, and respiratory syncytial virus infections in a large US integrated healthcare population
Source: PLoS Comput Biol. 2025 Nov 19;21(11):e1013723. doi: 10.1371/journal.pcbi.1013723 (PMC12643285; doi:10.1371/journal.pcbi.1013723)
Supplement: S1 File — (ZIP) [file pcbi.1013723.s001.zip › S1 File/S2_Table.pdf]

S2 Table: Counts of patients with comorbidities included in Charlson comorbidity index.

Condition	Number of patients, n (%)
Myocardial infarction	2057 (2.5)
Congestive heart failure	4117 (4.9)
Peripheral vascular disease	13156 (15.8)
CEVD	2468 (3.0)
Dementia	1773 (2.1)
COPD	14201 (17.1)
Rheumatic disease	1651 (2.0)
Peptic ulcer disease	331 (0.4)
Diabetes with complications	8460 (10.2)
Paraplegic	587 (0.7)
Renal disease	7600 (9.1)
Mild/severe liver disease	281 (0.3)
Metastatic cancer	993 (1.2)
HIV	139 (0.2)
Diabetes without complications	7188 (8.6)
Mild liver disease	3906 (4.7)

All conditions are considered within a year of a patient’s index date.
