# Supplementary material for: Clinical progression parameters associated with SARS-CoV-2, influenza, and respiratory syncytial virus infections in a large US integrated healthcare population
Source: PLoS Comput Biol. 2025 Nov 19;21(11):e1013723. doi: 10.1371/journal.pcbi.1013723 (PMC12643285; doi:10.1371/journal.pcbi.1013723)
Supplement: S1 File — (ZIP) [file pcbi.1013723.s001.zip › S1 File/S3_Table.pdf]

S3 Table: Individual characteristics by infecting virus and severity threshold reached.

Characteristic		Cases, according to infecting virus and severity threshold reached, *n* (%)					
		Infections receiving ARI diagnoses in any setting			*Infections resulting in inpatient admission*		
		SARS-CoV-2	Influenza	RSV	SARS-CoV-2	Influenza	RSV
		N=40,537	*N*=18,928	*N*=1,550	*N*=4,772	*N*=1,174	*N*=558
Age, years							
	0-17	2,863 (7.3)	5,610 (29.6)	842 (54.3)	74 (1.6)	85 (7.2)	129 (23.1)
	18-49	12,674 (32.3)	6,506 (34.4)	65 (4.2)	427 (8.9)	181 (15.4)	28 (5.0)
	50-59	5,961 (15.2)	2,387 (12.6)	80 (5.2)	334 (7.0)	105 (8.9)	47 (8.4)
	60-69	6,380 (16.3)	2,080 (11.0)	138 (8.9)	693 (14.5)	208 (17.7)	78 (14.0)
	70-79	6,381 (16.3)	1,564 (8.3)	167 (10.8)	1,355 (28.4)	318 (27.1)	99 (17.7)
	80-89	3,937 (10.0)	640 (3.4)	187 (12.1)	1,370 (28.7)	210 (17.9)	127 (22.8)
	≥90	1,035 (2.6)	141 (0.7)	71 (4.6)	519 (10.9)	67 (5.7)	50 (9.0)
Sex							
	Male	15,823 (40.3)	8,557 (45.2)	716 (46.2)	2,377 (49.8)	535 (45.6)	234 (41.9)
	Female	23,408 (59.7)	10,371 (54.8)	834 (53.8)	2,395 (50.2)	639 (54.4)	324 (58.1)
Race/ethnicity							
	White, non-Hispanic	10,223 (26.1)	4,249 (22.4)	451 (29.1)	2,020 (42.3)	405 (34.5)	206 (36.9)
	Asian, non-Hispanic	4,403 (11.2)	2,001 (10.6)	190 (12.3)	490 (10.3)	142 (12.1)	66 (11.8)
	Black, non-Hispanic	4,494 (11.5)	1,792 (9.5)	154 (9.9)	612 (12.8)	135 (11.5)	56 (10.0)
	Hispanic (any race)	18,282 (46.6)	9,841 (52.0)	705 (45.5)	1,564 (32.8)	473 (40.3)	214 (38.4)
	Native American/Alaska native	87 (0.2)	27 (0.1)	3 (0.2)	16 (0.3)	4 (0.3)	3 (0.5)
	Other	528 (1.3)	313 (1.7)	11 (0.7)	15 (0.3)	3 (0.3)	2 (0.4)
	Multiple	128 (0.3)	90 (0.5)	8 (0.5)	10 (0.2)	1 (0.1)	1 (0.2)
	Pacific Islander	303 (0.8)	131 (0.7)	12 (0.8)	37 (0.8)	9 (0.8)	8 (1.4)
	Unknown	783 (2.0)	484 (2.6)	16 (1.0)	8 (0.2)	2 (0.2)	2 (0.4)
Insurance source							
	Commercial	18,057 (46.0)	10,425 (55.1)	634 (40.9)	823 (17.2)	314 (26.7)	143 (25.6)
	Medicaid	4,437 (11.3)	2,663 (14.1)	279 (18.0)	512 (10.7)	159 (13.5)	92 (16.5)
	Medicare	10,665 (27.2)	2,347 (12.4)	381 (24.6)	2,898 (60.7)	533 (45.4)	241 (43.2)
	Pre-paid plans	945 (2.4)	552 (2.9)	30 (1.9)	46 (1.0)	20 (1.7)	10 (1.8)
	Other	473 (1.2)	157 (0.8)	14 (0.9)	97 (2.0)	18 (1.5)	5 (0.9)
	Unknown	4,654 (11.9)	2,784 (14.7)	212 (13.7)	396 (8.3)	130 (11.1)	67 (12.0)
Vaccinations							
	1-2 COVID-19 vaccine doses	7,263 (18.5)	4,331 (22.9)	147 (9.5)	706 (14.8)	200 (17.0)	62 (11.1)
	3+ COVID-19 vaccine doses	27,174 (69.3)	9,467 (50.0)	682 (44.0)	3,542 (74.2)	798 (68.0)	371 (66.5)
	Seasonal influenza vaccine received	22,810 (71.1)	10,448 (55.2)	1201 (77.5)	4,028 (84.4)	898 (76.5)	468 (83.9)
	RSV vaccine received	339 (0.9)	188 (1.0)	26 (1.7)	68 (1.4)	28 (2.4)	12 (2.2)
Neighborhood deprivation index							
	NDI < –1	1,775 (4.4)	839 (4.4)	68 (4.4)	253 (5.3)	46 (3.9)	28 (5.0)
	–1 ≤ NDI <0	13,331 (32.9)	5,913 (31.2)	499 (32.2)	1,704 (35.7)	388 (33.0)	167 (29.9)
	0 ≤ NDI < 1	13,943 (34.4)	6,605 (34.9)	535 (34.5)	1,610 (33.7)	428 (36.5)	205 (36.7)
	NDI > 1	7,583 (18.7)	3,726 (19.7)	262 (16.9)	765 (16.0)	212 (18.1)	92 (16.5)
	Unknown	3,904 (9.6)	1,845 (9.7)	186 (12.0)	440 (9.2)	100 (8.5)	66 (11.8)
Charlson comorbidity index							
	0	19,220 (47.4)	11,969 (63.2)	763 (49.2)	602 (12.6)	226 (19.3)	120 (21.5)
	1-2	11,905 (29.4)	4,823 (25.5)	354 (22.8)	1,090 (22.8)	344 (29.3)	142 (25.4)
	3-5	6,042 (14.9)	1,397 (7.4)	243 (15.7)	1,582 (33.2)	332 (28.3)	154 (27.6)
	6+	3,369 (8.3)	739 (3.9)	190 (12.3)	1,498 (31.4)	272 (23.2)	142 (25.4)

ARI: Acute respiratory illness. Diagnosis codes used to identify healthcare encounters signifying clinical progression are listed in **S1 Table**.
