# Supplementary material for: Clinical progression parameters associated with SARS-CoV-2, influenza, and respiratory syncytial virus infections in a large US integrated healthcare population
Source: PLoS Comput Biol. 2025 Nov 19;21(11):e1013723. doi: 10.1371/journal.pcbi.1013723 (PMC12643285; doi:10.1371/journal.pcbi.1013723)
Supplement: S1 File — (ZIP) [file pcbi.1013723.s001.zip › S1 File/S4_Table.pdf]

S4 Table: Best-fitting distributions for care utilization pathways for all infections using a 20-day follow-up period.

Originating state	Next outcome	Best-fitting distribution	Parameter 1	Parameter 2	Parameter 3
SARS-CoV-2 infections					
Symptoms onset					
	Virtual care	Log normal	logmean = 1.11	logsd = 0.786	
	Outpatient office visit	Log normal	logmean = 1.29	logsd = 0.793	
	Urgent care	Log normal	logmean = 1.12	logsd = 0.746	
	Emergency department	Log normal	logmean = 1.26	logsd = 0.824	
	Inpatient admission	Weibull	shape = 1.48	scale = 6.89	
Receipt of test					
	Virtual care	Log normal	logmean = -0.701	logsd = 1.66	
	Outpatient office visit	Log normal	logmean = -2.14	logsd = 1.49	
	Urgent care	Log normal	logmean = -2.01	logsd = 0.761	
	Emergency department	Log normal	logmean = -2.17	logsd = 0.654	
	Inpatient admission	Generalized gamma	mu = -2.15	sigma = 0.785	Q = 0
Virtual care					
	Outpatient office visit	Gamma	shape = 0.585	rate = 0.107	
	Urgent care	Log normal	logmean = -0.817	logsd = 1.88	
	Emergency department	Log normal	logmean = -0.28	logsd = 1.73	
	Inpatient admission	Gamma	shape = 0.600	rate = 0.156	
Outpatient office visit					
	Urgent care	Log normal	logmean = -1.18	logsd = 1.72	
	Emergency department	Weibull	shape = 0.681	scale = 2.00	
	Inpatient admission	Weibull	shape = 0.581	scale = 1.50	
Urgent care					
	Emergency department	Log normal	logmean = -0.271	logsd = 1.79	
	Inpatient admission	Generalized gamma	mu = -0.860	sigma = 1.79	Q = 0
Emergency department					
	Inpatient admission	Log normal	logmean = -0.372	logsd = 1.83	
	Mechanical ventilation	Gompertz	shape = 1.18	rate = 0.218	
	Death	Gamma	shape = 0.712	rate = 0.105	
Inpatient admission					
	Mechanical ventilation	Gamma	shape = 0.649	rate = 0.136	
	Death	Generalized gamma	mu = 2.90	sigma = 0.108	Q = 8.62
Mechanical ventilation					
	Death	Gamma	shape = 0.602	rate = 0.110	
Influenza infections					
Symptoms onset					
	Virtual care	Log normal	logmean = 1.12	logsd = 0.772	
	Outpatient office visit	Log normal	logmean = 1.26	logsd = 0.754	
	Urgent care	Log normal	logmean = 1.09	logsd = 0.728	
	Emergency department	Log normal	logmean = 1.22	logsd = 0.759	
	Inpatient admission	Gamma	logmean = 1.53	logsd = 0.792	
Receipt of test					
	Virtual care	Log normal	logmean = -1.65	logsd = 1.370	
	Outpatient office visit	Log normal	logmean = -2.07	logsd = 0.909	
	Urgent care	Log normal	logmean = -2.27	logsd = 0.340	
	Emergency department	Log normal	logmean = -2.25	logsd = 0.375	
	Inpatient admission	Log normal	logmean = -2.22	logsd = 0.525	
Virtual care					
	Outpatient office visit	Gamma	shape = 0.645	rate = 0.148	
	Urgent care	Log normal	logmean = -0.364	logsd = 1.64	
	Emergency department	Generalized gamma	mu = 0.01	sigma = 1.53	Q = 0.513
	Inpatient admission	Exponential	rate = 0.387		
Outpatient office visit					
	Urgent care	Log normal	logmean = -1.19	logsd = 1,71	
	Emergency department	Weibull	shape = 0.680	scale = 2.00	
	Inpatient admission	Weibull	shape = 0.579	scale = 1.55	
Urgent care					
	Emergency department	Log normal	logmean = -0.406	logsd = 1.69	
	Inpatient admission	Log normal	logmean = -0.655	logsd = 1.86	
Emergency department					
	Inpatient admission	Log normal	logmean = -0.405	logsd = 1.73	
	Mechanical ventilation	Log normal	logmean = -0.304	logsd = 1.68	
	Death	Gompertz	shape = 0.125	rate = 0.0310	
Inpatient admission					
	Mechanical ventilation	Generalized gamma	mu = -0.181	sigma = 1.76	Q = 0.00
	Death	Gompertz	shape = 0.140	rate = 0.0426	
Mechanical ventilation					
	Death	Gamma	shape = 0.592	rate = 0.104	
RSV infections					
Symptoms onset					
	Virtual care	Gamma	shape = 2.10	rate = 0.330	
	Outpatient office visit	Gamma	shape = 2.12	rate = 0.393	
	Urgent care	Log normal	logmean = 1.40	logsd = 0.751	

	Emergency department	Log normal	logmean = 1.43	logsd = 0.703	
	Inpatient admission	Log normal	logmean = 1.56	logsd = 0.677	
Receipt of test					
	Virtual care	Gamma	shape = 0.534	rate = 0.121	
	Outpatient office visit	Log normal	logmean = -1.27	logsd = 1.72	
	Urgent care	Log normal	logmean = -2.07	logsd = 0.89	
	Emergency department	Generalized gamma	mu = -2.30	sigma = 0.00358	Q = -14.70
	Inpatient admission	Log normal	logmean = -2.04	logsd = 1.04	
Virtual care					
	Outpatient office visit	Gamma	shape = 0.665	rate = 0.125	
	Urgent care	Log normal	logmean = -0.803	logsd = 1.76	
	Emergency department	Weibull	shape = 0.713	scale = 1.99	
	Inpatient admission	Exponential	rate = 0.540		
Outpatient office visit					
	Urgent care	Gamma	shape = 0.479	rate = 0.120	
	Emergency department	Log normal	logmean = -0.252	logsd = 1.74	
	Inpatient admission	Log normal	logmean = -0.725	logsd = 1.88	
Urgent care					
	Emergency department	Log normal	logmean = -0.598	logsd = 1.57	
	Inpatient admission	Log normal	logmean = -1.02	logsd = 1.50	
Emergency department					
	Inpatient admission	Log normal	logmean = -0.472	logsd = 1.57	
	Mechanical ventilation	Gompertz	shape = 47.0	rate = 0.432	
	Death	Exponential	rate = 0.121		
Inpatient admission					
	Mechanical ventilation	Gamma	shape = 0.709	rate = 0.201	
	Death	Gompertz	shape = 0.231	rate = 0.0261	
Mechanical ventilation					
	Death	Gompertz	shape = 0.402	rate = 0.0267	
