# Supplementary material for: Clinical progression parameters associated with SARS-CoV-2, influenza, and respiratory syncytial virus infections in a large US integrated healthcare population
Source: PLoS Comput Biol. 2025 Nov 19;21(11):e1013723. doi: 10.1371/journal.pcbi.1013723 (PMC12643285; doi:10.1371/journal.pcbi.1013723)
Supplement: S1 File — (ZIP) [file pcbi.1013723.s001.zip › S1 File/S5_Table.pdf]

**S5 Table: Best-fitting distributions for care utilization pathways at each acuity threshold for each infection using a 60-day follow-up period.**

| Originating state | Highest-acuity outcome | Best-fitting distribution | Parameter 1 | Parameter 2 | Parameter 3 |
|---|---|---|---|---|---|
| <u>*SARS-CoV-2 infections*</u> | | | | | |
| Symptoms onset | | | | | |
| | Virtual care (*or higher*) | Log normal | logmean = 1.36 | logsd = 0.886 | |
| | Outpatient office visit (*or higher*) | Log normal | logmean = 1.43 | logsd = 0.934 | |
| | Urgent care (*or higher*) | Log normal | logmean = 1.40 | logsd = 0.911 | |
| | Emergency department (*or higher*) | Generalized gamma | mu = 1.47 | sigma = 0.928 | Q = -0.139 |
| | Inpatient admission (*or higher*) | Log normal | logmean = 1.93 | logsd = 0.971 | |
| | Mechanical ventilation (*or higher*) | Gompertz | shape = 0.0387 | rate = 0.0190 | |
| | Death | Generalized gamma | shape = 0.0487 | rate = 0.0128 | |
| Receipt of test | | | | | |
| | Virtual care (*or higher*) | Log normal | logmean = -1.58 | logsd = 1.56 | |
| | Outpatient office visit (*or higher*) | Log normal | logmean = -1.75 | logsd = 1.53 | |
| | Urgent care (*or higher*) | Log normal | logmean = -1.89 | logsd = 1.33 | |
| | Emergency department (*or higher*) | Log normal | logmean = -1.84 | logsd = 1.37 | |
| | Inpatient admission (*or higher*) | Log normal | logmean = -1.02 | logsd = 2.14 | |
| | Mechanical ventilation (*or higher*) | Gamma | shape = 0.900 | rate = 0.0462 | |
| | Death | Gompertz | shape = 0.0270 | rate = 0.0270 | |
| Virtual care | | | | | |
| | Outpatient office visit (*or higher*) | Gamma | shape = 0.431 | rate = 0.0398 | |
| | Urgent care (*or higher*) | Generalized gamma | mu = 0.892 | sigma = 2.09 | Q = 0.314 |
| | Emergency department (*or higher*) | Generalized gamma | mu = 0.856 | sigma = 2.04 | Q = 0.301 |
| | Inpatient admission (*or higher*) | Gamma | shape = 0.543 | rate = 0.0423 | |
| | Mechanical ventilation (*or higher*) | Gompertz | shape = 0.0306 | rate = 0.0227 | |
| | Death | Gompertz | shape = 0.0384 | rate = 0.0179 | |
| Outpatient office visit | | | | | |
| | Urgent care (*or higher*) | Log normal | logmean = 0.0985 | logsd = 2.36 | |
| | Emergency department (*or higher*) | Generalized gamma | mu = 1.37 | sigma = 2.03 | Q = 0.693 |
| | Inpatient admission (*or higher*) | Gamma | shape = 0.4580 | rate = 0.0393 | |
| | Mechanical ventilation (*or higher*) | Gamma | shape = 0.824 | rate = 0.0448 | |
| | Death | Gompertz | shape = 0.0237 | rate = 0.0299 | |
| Urgent care | | | | | |
| | Emergency department (*or higher*) | Log normal | logmean = 0.0297 | logsd = 2.10 | |
| | Inpatient admission (*or higher*) | Log normal | logmean = 0.124 | logsd = 2.28 | |
| | Mechanical ventilation (*or higher*) | Gompertz | shape = 0.0284 | rate = 0.0256 | |
| | Death | Gompertz | shape = 0.0447 | rate = 0.0157 | |
| Emergency department | | | | | |
| | Inpatient admission (*or higher*) | Log normal | logmean = 0.3273 | logsd = 2.2076 | |
| | Mechanical ventilation (*or higher*) | Gompertz | shape = 0.0207 | rate = 0.0329 | |
| | Death | Gompertz | shape = 0.0269 | rate = 0.0272 | |
| Inpatient admission | | | | | |
| | Mechanical ventilation (*or higher*) | Generalized gamma | mu = 3.21 | sigma = 0.794 | Q = 2.01 |
| | Death | Gamma | shape = 1.49 | rate = 0.0748 | |
| Mechanical ventilation | | | | | |
| | Death | Gamma | shape = 0.528 | rate = 0.0481 | |
| <u>*Influenza infections*</u> | | | | | |
| Symptoms onset | | | | | |
| | Virtual care (*or higher*) | Log normal | logmean = 1.22 | logsd = 0.791 | |
| | Outpatient office visit (*or higher*) | Log normal | logmean = 1.26 | logsd = 0.806 | |
| | Urgent care (*or higher*) | Log normal | logmean = 1.27 | logsd = 0.815 | |
| | Emergency department (*or higher*) | Generalized gamma | mu = 1.33 | sigma = 0.832 | Q = -0.192 |
| | Inpatient admission (*or higher*) | Generalized gamma | mu = 1.92 | sigma = 0.914 | Q = 0.131 |
| | Mechanical ventilation (*or higher*) | Weibull | shape = 1.28 | scale = 20.40 | |
| | Death | Weibull | rate = 1.80 | scale = 27.80 | |
| Receipt of test | | | | | |
| | Virtual care (*or higher*) | Log normal | logmean = -2.13 | logsd = 0.810 | |
| | Outpatient office visit (*or higher*) | Log normal | logmean = -2.15 | logsd = 0.805 | |
| | Urgent care (*or higher*) | Log normal | logmean = -2.15 | logsd = 0.781 | |
| | Emergency department (*or higher*) | Log normal | logmean = -2.05 | logsd = 0.995 | |
| | Inpatient admission (*or higher*) | Log normal | logmean = -1.17 | logsd = 1.98 | |
| | Mechanical ventilation (*or higher*) | Gamma | rate = 0.580 | rate = 0.0386 | |
| | Death | Exponential | rate = 0.0493 | | |
| Virtual care | | | | | |
| | Outpatient office visit (*or higher*) | Log normal | logmean = 0.477 | logsd = 2.00 | |
| | Urgent care (*or higher*) | Log normal | logmean = 0.204 | logsd = 1.96 | |
| | Emergency department (*or higher*) | Log normal | logmean = 0.339 | logsd = 1.93 | |
| | Inpatient admission (*or higher*) | Gamma | shape = 0.574 | rate = 0.0443 | |
| | Mechanical ventilation (*or higher*) | Exponential | rate = 0.0512 | | |
| | Death | Gamma | shape = 1.68 | rate = 0.0811 | |
| Outpatient office visit | | | | | |
| | Urgent care (*or higher*) | Log normal | logmean = -0.208 | logsd = 2.24 | |
| | Emergency department (*or higher*) | Log normal | logmean = 0.269 | logsd = 2.07 | |
| | Inpatient admission (*or higher*) | Gamma | shape = 0.395 | rate = 0.0418 | |
| | Mechanical ventilation (*or higher*) | Gamma | shape = 0.522 | rate = 0.0326 | |

| | | | | |
|---|---|---|---|---|
| | Death | Exponential | rate = 0.0455 | |
| Urgent care | | | | |
| | Emergency department (*or higher*) | Log normal | logmean = -0.194 | logsd = 1.94 |
| | Inpatient admission (*or higher*) | Weibull | shape = 0.506 | scale = 3.04 |
| | Mechanical ventilation (*or higher*) | Exponential | rate = 0.0561 | |
| | Death | Exponential | rate = 0.0431 | |
| Emergency department | | | | |
| | Inpatient admission (*or higher*) | Log normal | logmean = 0.348 | logsd = 2.19 |
| | Mechanical ventilation (*or higher*) | Gamma | shape = 0.812 | rate = 0.0402 |
| | Death | Gompertz | shape = 0.0326 | rate = 0.0209 |
| Inpatient admission | | | | |
| | Mechanical ventilation (*or higher*) | Gamma | shape = 0.497 | rate = 0.0403 |
| | Death | Exponential | rate = 0.0518 | |
| Mechanical ventilation | | | | |
| | Death | Gamma | shape = 0.538 | rate = 0.0471 |
| | | | | |
| *RSV infections* | | | | |
| Symptoms onset | | | | |
| | Virtual care (*or higher*) | Log normal | logmean = 1.55 | logsd = 0.777 |
| | Outpatient office visit (*or higher*) | Log normal | logmean = 1.57 | logsd = 0.780 |
| | Urgent care (*or higher*) | Log normal | logmean = 1.61 | logsd = 0.773 |
| | Emergency department (*or higher*) | Log normal | logmean = 1.65 | logsd = 0.765 |
| | Inpatient admission (*or higher*) | Log normal | logmean = 1.85 | logsd = 0.762 |
| | Mechanical ventilation (*or higher*) | Log normal | logmean = 2.59 | logsd = 0.811 |
| | Death | Weibull | shape = 1.90 | scale = 28.80 |
| Receipt of test | | | | |
| | Virtual care (*or higher*) | Log normal | logmean = -1.93 | logsd = 1.22 |
| | Outpatient office visit (*or higher*) | Log normal | logmean = -1.97 | logsd = 1.16 |
| | Urgent care (*or higher*) | Log normal | logmean = -2.05 | logsd = 0.982 |
| | Emergency department (*or higher*) | Log normal | logmean = -2.01 | logsd = 1.04 |
| | Inpatient admission (*or higher*) | Log normal | logmean = -1.45 | logsd = 1.64 |
| | Mechanical ventilation (*or higher*) | Gamma | shape = 0.596 | rate = 0.0487 |
| | Death | Exponential | rate = 0.0512 | |
| Virtual care | | | | |
| | Outpatient office visit (*or higher*) | Weibull | shape = 0.554 | scale = 7.061 |
| | Urgent care (*or higher*) | Log normal | logmean = 0.536 | logsd = 2.016 |
| | Emergency department (*or higher*) | Log normal | logmean = 0.589 | logsd = 1.941 |
| | Inpatient admission (*or higher*) | Log normal | logmean = 1.19 | logsd = 1.85 |
| | Mechanical ventilation (*or higher*) | Exponential | rate = 0.0432 | |
| | Death | Exponential | rate = 0.0400 | |
| Outpatient office visit | | | | |
| | Urgent care (*or higher*) | Log normal | logmean = 0.0756 | logsd = 2.12 |
| | Emergency department (*or higher*) | Log normal | logmean = 0.0311 | logsd = 2.11 |
| | Inpatient admission (*or higher*) | Log normal | logmean = 0.213 | logsd = 2.25 |
| | Mechanical ventilation (*or higher*) | Gamma | shape = 0.636 | rate = 0.0411 |
| | Death | Exponential | rate = 0.0405 | |
| Urgent care | | | | |
| | Emergency department (*or higher*) | Log normal | logmean = -0.628 | logsd = 1.70 |
| | Inpatient admission (*or higher*) | Log normal | logmean = -0.651 | logsd = 1.68 |
| | Mechanical ventilation (*or higher*) | Exponential | rate = 0.0758 | |
| | Death | NA | NA | |
| Emergency department | | | | |
| | Inpatient admission (*or higher*) | Log normal | logmean = -0.0549 | logsd = 1.93 |
| | Mechanical ventilation (*or higher*) | Exponential | rate = 0.0650 | |
| | Death | Exponential | rate = 0.0618 | |
| Inpatient admission | | | | |
| | Mechanical ventilation (*or higher*) | Gamma | shape = 0.608 | rate = 0.0548 |
| | Death | Gamma | shape = 1.75 | rate = 0.0802 |
| Mechanical ventilation | | | | |
| | Death | Exponential | rate = 0.058 | |
