# Supplementary material for: Clinical progression parameters associated with SARS-CoV-2, influenza, and respiratory syncytial virus infections in a large US integrated healthcare population
Source: PLoS Comput Biol. 2025 Nov 19;21(11):e1013723. doi: 10.1371/journal.pcbi.1013723 (PMC12643285; doi:10.1371/journal.pcbi.1013723)
Supplement: S1 File — (ZIP) [file pcbi.1013723.s001.zip › S1 File/S6_Table.pdf]

S6 Table: Care utilization pathways associated with each infecting virus using a follow-up period of 60 days.

Originating state	Next outcome	Probability of progression, % (95% CI)	Time to progression along indicated transition pathway, days (95% CI)		
			Median	*25%ile*	*75%ile*
SARS-CoV-2 infections					
Symptoms onset					
	Virtual care	10.7 (10.5, 10.9)	3.08 (3.01, 3.15)	1.78 (1.74, 1.83)	5.32 (5.18, 5.46)
	Outpatient office visit	7.4 (7.3, 7.6)	3.66 (3.01, 3.15)	2.11 (2.05, 2.16)	6.66 (6.46, 6.87)
	Urgent care	18.6 (18.2, 19.0)	3.09 (3.02, 3.16)	1.85 (1.81, 1.90)	5.16 (5.03, 5.31)
	Emergency department	17.9 (17.6, 18.3)	3.54 (3.46, 3.63)	2.02 (1.96, 2.07)	6.21 (6.05, 6.38)
	Inpatient admission	4.7 (4.6, 4.8)	5.27 (5.09, 5.47)	2.89 (2.76, 3.02)	8.73 (8.46, 9.02)
	Mechanical ventilation	0.0 (0.0, 0.0)	2.20 (1.31, 3.70)	1.24 (0.67, 2.20)	3.93 (2.21, 7.07)
	Death	0.1 (0.1, 0.1)	7.78 (5.80, 10.41)	3.99 (2.96, 5.38)	15.17 (11.31, 20.53)
Receipt of test					
	Virtual care	14.3 (14.1, 14.5)	0.58 (0.57, 0.59)	<0.2	1.98 (1.94, 2.02)
	Outpatient office visit	7.9 (7.8, 8.0)	0.32 (0.32, 0.33)	<0.2	1.34 (1.31, 1.37)
	Urgent care	18.3 (18.1, 18.5)	<0.2	<0.2	0.29 (0.28, 0.29)
	Emergency department	19.8 (19.6, 20.0)	<0.2	<0.2	0.22 (0.21, 0.22)
	Inpatient admission	4.7 (4.6, 4.8)	<0.2	<0.2	<0.2
	Mechanical ventilation	0.0 (0.0, 0.1)	4.11 (1.98, 8.72)	0.91 (0.30, 2.50)	13.52 (7.21, 24.33)
	Death	0.2 (0.2, 0.2)	14.71 (11.58, 17.94)	6.45 (4.96, 8.25)	27.03 (22.11, 33.10)
Virtual care					
	Outpatient office visit	10.7 (10.4, 10.9)	7.63 (7.29, 8.00)	1.88 (1.76, 2.02)	20.98 (20.28, 21.75)
	Urgent care	6.1 (5.9, 6.2)	2.07 (1.79, 2.37)	0.44 (0.39, 0.48)	8.48 (7.77, 9.52)
	Emergency department	7.8 (7.6, 7.9)	0.96 (0.86, 1.07)	0.27 (0.25, 0.29)	3.78 (3.56, 4.09)
	Inpatient admission	2.3 (2.2, 2.3)	3.30 (3.22, 3.39)	0.74 (0.70, 0.78)	10.68 (10.39, 10.94)
	Mechanical ventilation	0.0 (0.0, 0.1)	16.24 (9.10, 27.64)	10.19 (5.39, 18.51)	25.87 (15.34, 50.13)
	Death	0.3 (0.2, 0.4)	20.46 (17.07, 24.40)	11.99 (8.55, 15.80)	31.18 (28.90, 33.95)
Outpatient office visit					
	Urgent care	11.1 (11.0, 11.2)	0.54 (0.48, 0.64)	0.11 (0.10, 0.13)	2.66 (2.28, 3.10)
	Emergency department	8.6 (8.5, 8.7)	1.64 (1.41, 1.93)	0.37 (0.31, 0.43)	7.31 (6.22, 8.52)
	Inpatient admission	4.8 (4.7, 4.8)	1.59 (1.28, 2.00)	0.34 (0.27, 0.43)	7.40 (5.96, 9.37)
	Mechanical ventilation	0.5 (0.4, 0.6)	1.10 (0.59, 1.99)	0.29 (0.15, 0.56)	4.08 (2.08, 8.49)
	Death	1.2 (0.1, 1.4)	13.52 (10.34, 17.00)	5.85 (4.46, 7.71)	25.37 (20.60, 30.71)
Urgent care					
	Emergency department	7.5 (7.3, 7.6)	1.03 (0.90, 1.17)	0.27 (0.25, 0.29)	4.32 (4.02, 4.79)
	Inpatient admission	1.9 (1.8, 1.9)	1.48 (1.40, 1.56)	<0.2	6.45 (5.65, 7.36)
	Mechanical ventilation	0.0 (0.0, 0.1)	6.93 (1.79, 27.12)	2.88 (0.78, 12.31)	13.86 (3.64, 59.31)
	Death	0.0 (0.0, 0.1)	16.93 (6.50, 46.51)	7.03 (2.80, 18.33)	33.87 (13.48, 88.32)
Emergency department					
	Inpatient admission	8.6 (8.5, 8.7)	0.51 (0.49, 0.52)	<0.2	2.75 (2.51, 2.88)
	Mechanical ventilation	0.1 (0.1, 0.2)	6.74 (1.91, 16.60)	1.16 (0.10, 4.89)	22.65 (9.35, 51.49)
	Death	0.9 (0.7, 0.1)	11.85 (11.50, 12.17)	3.80 (3.15, 4.39)	27.83 (25.06, 30.7)
Inpatient admission					
	Mechanical ventilation	5.9 (5.8, 6.1)	3.82 (3.74, 3.91)	1.00 (0.96, 1.04)	11.01 (10.77, 11.24)
	Death	11.3 (11.1, 11.5)	17.35 (15.01, 20.49)	8.08 (7.49, 8.80)	29.59 (23.94, 39.92)
Mechanical ventilation					
	Death	50.2 (44.3, 55.6)	5.24 (4.95, 5.48)	1.25 (1.00, 1.47)	14.66 (12.86, 16.40)
Influenza infections					
Symptoms onset					
	Virtual care	8.2 (8.0, 8.3)	2.91 (2.86, 2.96)	1.77 (1.73, 1.80)	5.10 (4.99, 5.22)
	Outpatient office visit	12.8 (12.5, 13.0)	3.61 (3.55, 3.68)	2.13 (2.08, 2.217)	6.14 (6.02, 6.27)
	Urgent care	37.0 (36.6, 37.5)	3.02 (2.97, 3.07)	1.83 (1.79, 1.87)	4.99 (4.89, 5.09)
	Emergency department	28.6 (28.2, 29.1)	3.33 (3.27, 3.39)	2.01 (1.97, 2.05)	5.64 (5.52, 5.84)
	Inpatient admission	3.4 (3.3, 3.5)	5.17 (5.02, 5.31)	2.89 (2.80, 2.98)	8.44 (8.24, 8.64)
	Mechanical ventilation	0 (*not observed*)	– –	– –	– –
	Death	0.0 (0.0, 0.1)	5.55 (2.43, 12.87)	2.30 (1.01, 5.54)	11.09 (4.86, 26.72)
Receipt of test					
	Virtual care	7.0 (6.8, 7.1)	<0.2	<0.2	0.30 (0.29, 0.31)
	Outpatient office visit	11.9 (11.7, 12.1)	<0.2	<0.2	0.33 (0.32, 0.33)
	Urgent care	38.9 (38.4, 39.4)	<0.2	<0.2	<0.2
	Emergency department	31.2 (30.7, 31.7)	<0.2	<0.2	<0.2
	Inpatient admission	3.1 (3.1, 3.2)	<0.2	<0.2	<0.2
	Mechanical ventilation	0.0 (0.0, 0.1)	4.93 (2.48, 10.03)	2.05 (0.99, 3.89)	9.86 (4.79, 18.76)
	Death	0.1 (0.0, 0.1)	7.63 (2.78, 16.64)	2.00 (0.37, 5.86)	20.23 (9.97, 37.94)
Virtual care					
	Outpatient office visit	15.9 (15.8, 16.1)	5.28 (5.11, 5.45)	1.21 (1.09, 1.33)	15.11 (14.15, 16.15)
	Urgent care	12.5 (12.4, 12.7)	1.03 (0.86, 1.21)	0.28 (0.23, 0.33)	3.83 (3.19, 4.55)
	Emergency department	12.4 (12.3, 12.5)	1.23 (1.03, 1.48)	0.26 (0.21, 0.32)	4.22 (3.59, 4.91)
	Inpatient admission	1.6 (1.3, 2.1)	6.69 (5.20, 8.63)	2.78 (2.15, 3.57)	13.38 (10.38, 17.20)
	Mechanical ventilation	0.0 (0.0, 0.2)	– –	– –	– –
	Death	0.1 (0.0, 0.3)	29.20 (21.30, 41.20)	23.22 (15.48, 33.35)	36.85 (26.10, 55.55)
Outpatient office visit					
	Urgent care	15.9 (15.7, 16.0)	0.56 (0.48, 0.67)	<0.2	2.62 (2.21, 3.10)
	Emergency department	8.7 (8.6, 8.8)	1.33 (1.09, 1.63)	0.34 (0.28, 0.41)	5.19 (4.24, 6.29)

	Inpatient admission	2.6 (2.2, 3.1)	1.20 (0.80, 1.85)	0.25 (0.17, 0.39)	5.65 (3.78, 8.59)
	Mechanical ventilation	0.2 (0.1, 0.4)	0.56 (0.16, 1.77)	0.17 (0.04, 0.61)	1.92 (0.51, 7.84)
	Death	0.4 (0.3, 0.6)	7.55 (4.16, 12.9)	3.12 (1.58, 5.88)	18.23 (9.65, 35.48)
Urgent care					
	Emergency department	7.8 (7.7, 7.9)	0.85 (0.84, 0.87)	0.23 (0.23, 0.24)	3.10 (3.00, 3.20)
	Inpatient admission	1.4 (1.3, 1.4)	0.66 (0.45, 0.95)	<0.2	2.67 (1.88, 3.86)
	Mechanical ventilation	0 (*not observed*)	– –	– –	– –
	Death	0.0 (0.0, 0.1)	3.98 (0.00, 4.08)	3.92 (0.00, 4.03)	4.02 (0.00, 4.15)
Emergency department					
	Inpatient admission	4.1 (4.1, 4.2)	1.21 (0.96, 1.53)	0.29 (0.23, 0.36)	5.15 (4.08, 6.50)
	Mechanical ventilation	0.1 (0.1, 0.2)	1.61 (0.29, 6.25)	0.27 (0.02, 1.89)	6.62 (1.70, 26.96)
	Death	0.3 (0.2, 0.4)	26.05 (15.88, 32.51)	14.91 (7.74, 22.21)	37.05 (28.01, 43.62)
Inpatient admission					
	Mechanical ventilation	6.8 (5.5, 8.5)	1.10 (0.07, 1.13)	0.30 (0.29, 0.31)	7.82 (5.39, 10.88)
	Death	5.5 (4.4, 7.0)	17.50 (2.44, 23.52)	4.06 (3.91, 4.22)	31.38 (23.53, 44.89)
Mechanical ventilation					
	Death	34.5 (25.4, 45.7)	5.52 (2.87, 9.58)	1.35 (0.40, 3.19)	15.28 (9.02, 24.68)
RSV infections					
Symptoms onset					
	Virtual care	6.2 (5.8, 6.6)	5.15 (5.06, 5.25)	2.99 (2.93, 3.06)	8.87 (8.68, 9.06)
	Outpatient office visit	12.9 (12.5, 13.3)	4.37 (4.29, 4.45)	2.54 (2.49, 2.6)	7.51 (7.35, 7.68)
	Urgent care	10.8 (10.4, 11.2)	4.05 (3.79, 4.32)	2.42 (2.29, 2.50)	7.06 (6.85, 7.44)
	Emergency department	37.9 (37.3, 38.4)	4.19 (4.11, 4.28)	2.60 (2.55, 2.66)	6.76 (6.61, 6.9)
	Inpatient admission	18.7 (18.2, 19.1)	4.74 (4.65, 4.83)	3.00 (2.94, 3.06)	7.49 (7.33, 7.66)
	Mechanical ventilation	0 (*not observed*)	– –	– –	– –
	Death	0.1 (0.0, 0.4)	– –	– –	– –
Receipt of test					
	Virtual care	6.4 (5.4, 7.7)	2.97 (2.83, 3.08)	0.60 (0.48, 0.73)	0.6 (0.48, 0.73)
	Outpatient office visit	19.1 (18.6, 19.6)	<0.2	<0.2	<0.2
	Urgent care	9.3 (8.5, 10.0)	<0.2	<0.2	<0.2
	Emergency department	36.2 (35.7, 36.7)	<0.2	<0.2	<0.2
	Inpatient admission	14.4 (13.9, 14.8)	<0.2	<0.2	<0.2
	Mechanical ventilation	0.7 (0.4, 1.2)	2.10 (0.70, 6.24)	0.62 (0.19, 1.93)	0.62 (0.19, 1.93)
	Death	0.3 (0.1, 0.7)	2.77 (1.15, 6.79)	1.15 (0.46, 2.70)	1.15 (0.46, 2.7)
Virtual care					
	Outpatient office visit	25.6 (21.5, 30.0)	10.80 (8.24, 32.82)	1.95 (1.11, 15.77)	29.36 (26.36, 50.83)
	Urgent care	6.7 (4.5, 9.9)	1.28 (0.50, 3.58)	0.24 (0.08, 0.63)	6.82 (2.45, 20.16)
	Emergency department	16.9 (13.4, 21.3)	1.36 (0.87, 2.11)	0.38 (0.23, 0.58)	4.95 (3.04, 7.64)
	Inpatient admission	4.6 (2.9, 7.4)	1.08 (0.50, 2.55)	0.34 (0.15, 0.78)	3.42 (1.45, 8.42)
	Mechanical ventilation	0 (*not observed*)			
	Death	0.8 (0.2, 2.3)	17.41 (5.48, 51.70)	7.23 (2.41, 20.80)	34.82 (11.63, 100.22)
Outpatient office visit					
	Urgent care	7.9 (6.0, 10.1)	2.25 (1.26, 3.94)	0.44 (0.20, 1.04)	8.1 (5.07, 13.53)
	Emergency department	18.2 (15.4, 21.1)	1.15 (0.81, 1.67)	0.30 (0.20, 0.41)	4.51 (3.14, 6.33)
	Inpatient admission	11.8 (9.7, 14.5)	0.63 (0.40, 0.99)	<0.2	2.57 (1.54, 4.32)
	Mechanical ventilation	1.0 (0.5, 2.2)	1.78 (0.31, 6.85)	0.33 (0.01, 2.09)	5.73 (1.69, 17.28)
	Death	1.3 (0.7, 2.5)	11.56 (6.35, 21.72)	4.80 (2.48, 9.07)	23.12 (12.31, 44.71)
Urgent care					
	Emergency department	30.2 (25.4, 34.7)	0.62 (0.45, 0.85)	0.20 (0.14, 0.28)	1.97 (1.42, 2.79)
	Inpatient admission	17.7 (14.1, 21.9)	0.39 (0.26, 0.57)	<0.2	1.13 (0.76, 1.7)
	Mechanical ventilation	0.3 (0.0, 2.0)	11.78 (1.74, 85.28)	4.89 (0.71, 34.47)	23.57 (3.4, 66.09)
	Death				
Emergency department					
	Inpatient admission	17.6 (17.3, 17.8)	0.87 (0.67, 1.17)	0.24 (0.18, 0.34)	3.11 (2.34, 4.27)
	Mechanical ventilation	0.1 (0.0, 0.8)	0.07 (0.01, 0.49)	<0.2	0.14 (0.02, 0.85)
	Death	0.7 (0.3, 1.5)	13.40 (6.02, 28.53)	5.56 (2.44, 11.73)	26.8 (11.73, 56.55)
Inpatient admission					
	Mechanical ventilation	7.4 (5.5, 10.0)	3.28 (1.87, 5.30)	0.84 (0.34, 1.74)	8.81 (5.96, 13.23)
	Death	3.5 (2.1, 5.4)	18.68 (11.35, 26.37)	9.21 (4.8, 15.68)	30.06 (21.22, 38.01)
Mechanical ventilation					
	Death	20.0 (10.0, 37.1)	11.96 (5.77, 22.86)	4.96 (2.43, 10.48)	23.91 (11.71, 50.51)

We indicate values as <0.2 where maximum likelihood estimates and accompanying confidence limits fell below 0.2 days, which arose in scenarios where a vast majority of transitions along the indicated path were observed occurring on the same day. We report estimates from best-fitting distributions, based on models yielding the minimum AIC score; we indicate these distributions and their parameters parameter in **S7 Table**.
