# Supplementary material for: Clinical progression parameters associated with SARS-CoV-2, influenza, and respiratory syncytial virus infections in a large US integrated healthcare population
Source: PLoS Comput Biol. 2025 Nov 19;21(11):e1013723. doi: 10.1371/journal.pcbi.1013723 (PMC12643285; doi:10.1371/journal.pcbi.1013723)
Supplement: S1 File — (ZIP) [file pcbi.1013723.s001.zip › S1 File/S7_TAble.pdf]

S7 Table: Best-fitting distributions for care utilization pathways for all infections using a 60-day follow-up period.

Originating state	Next outcome	Best-fitting distribution	Parameter 1	Parameter 2	Parameter 3
SARS-CoV-2 infections					
Symptoms onset					
	Virtual care	Log normal	logmean = 1.13	logsd = 0.810	
	Outpatient office visit	Generalized gamma	mu = 1.20	sigma = 0.836	Q = -0.361
	Urgent care	Log normal	logmean = 1.13	logsd = 0.761	
	Emergency department	Log normal	logmean = 1.26	logsd = 0.833	
	Inpatient admission	Gamma	shape = 1.77	rate = 0.275	
	Mechanical ventilation	Log normal	logmean = 0.79	logsd = 0.857	
	Death	Log normal	logmean = 2.05	logsd = 0.991	
Receipt of test					
	Virtual care	Log normal	logmean = -0.543	logsd = 1.82	
	Outpatient office visit	Log normal	logmean = -1.13	logsd = 2.11	
	Urgent care	Log normal	logmean = -2.01	logsd = 1.14	
	Emergency department	Log normal	logmean = -2.11	logsd = 0.867	
	Inpatient admission	Generalized gamma	mu = -2.30	sigma = 0.0142	Q = -17.8
	Mechanical ventilation	Weibull	shape = 0.583	rate = 7.72	
	Death	Gompertz	shape = 0.0131	rate = 0.0427	
Virtual care					
	Outpatient office visit	Gamma	shape = 0.590	rate = 0.0346	
	Urgent care	Generalized gamma	mu = 1.01	sigma = 2.14	Q = 0.395
	Emergency department	Generalized gamma	mu = -0.237	sigma = 1.94	Q = -0.296
	Inpatient admission	Weibull	shape = 0.590	rate = 6.14	
	Mechanical ventilation	Log normal	logmean = 2.79	logsd = 0.691	
	Death	Weibull	shape = 1.65	rate = 25.6	
Outpatient office visit					
	Urgent care	Log normal	logmean = 0.607	logsd = 2.35	
	Emergency department	Log normal	logmean = 0.495	logsd = 2.21	
	Inpatient admission	Weibull	logmean = 0.466	logsd = 2.28	
	Mechanical ventilation	Log normal	logmean = 0.0913	logsd = 1.95	
	Death	Gompertz	shape = 0.0105	rate = 0.0477	
Urgent care					
	Emergency department	Generalized gamma	mu = -0.173	sigma = 2.03	Q = -0.296
	Inpatient admission	Gamma	shape = 0.319	rate = 0.0581	
	Mechanical ventilation	Exponential			
	Death	Exponential	rate = 0.0409		
Emergency department					
	Inpatient admission	Generalized gamma	mu = -2.29	sigma = 0.188	Q = -12.90
	Mechanical ventilation	Gamma	shape = 0.416	rate = 0.0234	
	Death	Gamma	shape = 0.706	rate = 0.0349	
Inpatient admission					
	Mechanical ventilation	Weibull	shape = 0.655	scale = 6.69	
	Death	Gompertz	shape = 0.0238	rate = 0.0323	
Mechanical ventilation					
	Death	Gamma	shape = 0.528	rate = 0.0481	
Influenza infections					
Symptoms onset					
	Virtual care	Generalized gamma	mu = 0.932	sigma = 0.755	Q = -0.521
	Outpatient office visit	Log normal	logmean = 1.29	logsd = 0.786	
	Urgent care	Log normal	logmean = 1.11	logsd = 0.744	
	Emergency department	Generalized gamma	mu = 0.940	sigma = 0.707	Q = -0.440
	Inpatient admission	Gamma	shape = 1.87	rate = 0.299	
	Mechanical ventilation	*No events observed*			
	Death	Exponential	rate = 0.125		
Receipt of test					
	Virtual care	Generalized gamma	mu = -2.30	sigma = 0.0537	Q = -15.51
	Outpatient office visit	Log normal	logmean = -1.95	logsd = 1.22	
	Urgent care	Log normal	logmean = -2.25	logsd = 0.476	
	Emergency department	Log normal	logmean = -2.24	logsd = 0.454	
	Inpatient admission	Log normal	logmean = -2.17	logsd = 0.757	
	Mechanical ventilation	Exponential	rate = 0.141		
	Death	Gamma	shape = 0.573	rate = 0.0382	
Virtual care					
	Outpatient office visit	Gamma	shape = 0.512	rate = 0.0450	
	Urgent care	Log normal	logmean = 0.0110	logsd = 1.95	
	Emergency department	Weibull	shape = 0.563	scale = 2.36	
	Inpatient admission	Exponential	rate = 0.104		
	Mechanical ventilation	Gompertz	shape = 0.149	rate = 0.0000792	
	Death	Log normal	logmean = 3.38	logsd = 0.342	
Outpatient office visit					
	Urgent care	Log normal	logmean = -0.597	logsd = 2.27	
	Emergency department	Log normal	logmean = 0.284	logsd = 2.02	
	Inpatient admission	Log normal	logmean = 0.199	logsd = 2.30	
	Mechanical ventilation	Log normal	logmean = -0.567	logsd = 1.82	
	Death	Log normal	logmean = 2.02	logsd = 1.31	

Urgent care					
	Emergency department	Log normal	logmean = -0.159	logsd = 1.91	
	Inpatient admission	Log normal	logmean = -0.409	logsd = 2.06	
	Mechanical ventilation	*No events observed*			
	Death	Gompertz	shape = 0.160	rate = 0.00	
Emergency department					
	Inpatient admission	Log normal	logmean = 0.194	logsd = 2.14	
	Mechanical ventilation	Weibull	shape = 0.492	scale = 3.39	
	Death	Gompertz	shape = 0.0496	rate = 0.0130	
Inpatient admission					
	Mechanical ventilation	Generalized gamma	mu = 0.095	sigma = 1.94	Q = 0.00
	Death	Gompertz	shape = 0.0148	rate = 0.0347	
Mechanical ventilation					
	Death	Gamma	shape = 0.538	rate = 0.0471	
RSV infections					
Symptoms onset					
	Virtual care	Log normal	logmean = 1.64	logsd = 0.807	
	Outpatient office visit	Log normal	logmean = 1.47	logsd = 0.802	
	Urgent care	Generalized gamma	mu = 1.31	sigma = 0.781	Q = -0.334
	Emergency department	Log normal	logmean = 1.43	logsd = 0.707	
	Inpatient admission	Log normal	logmean = 1.56	logsd = 0.677	
	Mechanical ventilation	*No events observed*			
	Death	Gompertz	shape = 0.115	rate = 0.00192	
Receipt of test					
	Virtual care	Gamma	shape = 0.465	rate = 0.0666	
	Outpatient office visit	Generalized gamma	mu = -2.30	sigma = 0.0967	Q = -13.70
	Urgent care	Log normal	logmean = -2.00	logsd = 1.10	
	Emergency department	Generalized gamma	mu = -2.30	sigma = 0.00863	Q = -11.60
	Inpatient admission	Generalized gamma	mu = -2.30	sigma = 0.0235	Q = -13.20
	Mechanical ventilation	Log normal	logmean = 0.744	logsd = 1.82	
	Death	Exponential	rate = 0.250		
Virtual care					
	Outpatient office visit	Generalized gamma	mu = 3.97	sigma = 0.274	Q = 8.99
	Urgent care	Log normal	logmean = 0.248	logsd = 2.48	
	Emergency department	Log normal	logmean = 0.310	logsd = 1.91	
	Inpatient admission	Log normal	logmean = 0.0725	logsd = 1.71	
	Mechanical ventilation	*No events observed*			
	Death	Exponential	rate = 0.0398		
Outpatient office visit					
	Urgent care	Weibull	shape = 0.541	scale = 4.43	
	Emergency department	Log normal	logmean = 0.144	logsd = 2.02	
	Inpatient admission	Log normal	logmean = -0.466	logsd = 2.09	
	Mechanical ventilation	Log normal	logmean = 0.438	logsd = 0.0987	
	Death	Exponential	rate = 0.0600		
Urgent care					
	Emergency department	Log normal	logmean = -0.474	logsd = 1.71	
	Inpatient admission	Log normal	logmean = -0.950	logsd = 1.59	
	Mechanical ventilation	*No events observed*			
	Death	Exponential	rate = 0.0588		
Emergency department					
	Inpatient admission	Log normal	logmean = -0.140	logsd = 1.89	
	Mechanical ventilation	Exponential	rate = 9.98		
	Death	Exponential	rate = 0.0517		
Inpatient admission					
	Mechanical ventilation	Gamma	shape = 0.562	rate = 0.0858	
	Death	Gompertz	shape = 0.0337	rate = 0.0266	
Mechanical ventilation					
	Death	Exponential	rate = 0.058		
