# Supplementary material for: Clinical progression parameters associated with SARS-CoV-2, influenza, and respiratory syncytial virus infections in a large US integrated healthcare population
Source: PLoS Comput Biol. 2025 Nov 19;21(11):e1013723. doi: 10.1371/journal.pcbi.1013723 (PMC12643285; doi:10.1371/journal.pcbi.1013723)
Supplement: S1 File — (ZIP) [file pcbi.1013723.s001.zip › S1 File/S8_Table.pdf]

S8 Table: Hospital length of stay estimates for admissions leading to discharge or mortality.

Infection	Outcome	Probability, % (95% CI)	Time to progression along indicated transition pathway, days (95% CI)		
			Median	*25%ile*	*75%ile*
SARS-CoV-2 infections					
	Any outcome	– –	4.24 (4.14, 4.33)	2.59 (2.53, 2.64)	7.27 (7.09, 7.46)
	Discharged alive	93.1 (92.4, 93.7)	4.11 (4.03, 4.20)	2.55 (2.50, 2.61)	6.94 (6.78, 7.12)
	In-hospital mortality	6.9 (6.3, 7.7)	7.25 (6.47, 8.10)	3.65 (3.21, 4.17)	13.3 (12.05, 14.68)
Influenza infections					
	Any outcome	– –	3.95 (3.78, 4.13)	2.30 (2.19, 2.42)	6.79 (6.47, 7.15)
	Discharged alive	95.9 (94.7, 96.9)	3.91 (3.73, 4.09)	2.30 (2.18, 2.42)	6.65 (6.32, 6.99)
	In-hospital mortality	4.1 (3.1, 5.3)	5.24 (3.93, 7.03)	2.60 (1.83, 3.56)	10.5 (7.74, 14.75)
RSV infections					
	Any outcome	– –	4.33 (4.06, 4.61)	2.54 (2.37, 2.72)	7.39 (6.89, 7.91)
	Discharged alive	98.4 (96.9, 99.0)	4.28 (4.01, 4.55)	2.52 (2.35, 2.70)	7.26 (6.77, 7.76)
	In-hospital mortality	1.6 (1.0, 3.1)	11.3 (5.41, 16.51)	5.80 (2.20, 10.46)	17.6 (10.16, 23.71)

We report estimates from best-fitting distributions, based on models yielding the minimum AIC score.
