# Supplementary material for: Clinical progression parameters associated with SARS-CoV-2, influenza, and respiratory syncytial virus infections in a large US integrated healthcare population
Source: PLoS Comput Biol. 2025 Nov 19;21(11):e1013723. doi: 10.1371/journal.pcbi.1013723 (PMC12643285; doi:10.1371/journal.pcbi.1013723)
Supplement: S1 File — (ZIP) [file pcbi.1013723.s001.zip › S1 File/S9_Table.pdf]

S9 Table: Proportions of cases attaining or exceeding each acuity threshold, by age.

Acuity threshold	Stratum	SARS-CoV-2 infections		Influenza infections		RSV infections	
		Proportion, % (95% CI)	*Median time from symptoms onset to event, days (95% CI)*	*Proportion, % (95% CI)*	*Median time from symptoms onset to event, days (95% CI)*	*Proportion, % (95% CI)*	*Median time from symptoms onset to event, days (95% CI)*
Virtual care (or higher)							
	0-17y	69.0 (67.5, 70.5)	3.16 (2.32, 4.26)	91.7 (91.2, 92.2)	3.12 (1.31, 7.24)	91.1 (88.9, 92.9)	– –
	18-49y	58.8 (58.1, 59.4)	3.73 (2.45, 5.73)	92.5 (92.3, 92.7)	3.20 (0.89, 10.74)	87.3 (77.5, 93.2)	– –
	50-59y	68.4 (67.3, 69.3)	3.77 (2.50, 5.86)	93.3 (93.1, 93.6)	3.41 (0.98, 12.18)	94.0 (86.5, 97.5)	– –
	60-69y	76.0 (75.1, 76.9)	4.00 (2.60, 6.48)	95.7 (95.4, 96.0)	3.79 (1.13, 12.56)	93.0 (88.0, 96.8)	– –
	70-79y	83.8 (83.0, 84.6)	4.28 (2.65, 6.69)	95.9 (95.1, 96.6)	4.27 (1.22, 14.64)	94.3 (89.2, 96.9)	– –
	80-89y	88.4 (87.4, 89.2)	4.38 (2.88, 6.73)	94.4 (92.3, 95.9)	4.27 (1.27, 15.54)	96.0 (93.0, 98.6)	– –
	≥90y	90.9 (88.9, 92.4)	4.45 (2.90, 6.70)	97.2 (92.9, 98.8)	4.32 (1.24, 13.91)	94.6 (86.9, 97.9)	– –
Outpatient office visit (or higher)							
	0-17y	58.9 (57.5, 60.3)	3.31 (2.46, 4.32)	87.8 (87.5, 88.2)	3.20 (1.45, 7.27)	88.1 (85.7, 90.1)	– –
	18-49y	41.2 (40.6, 41.8)	3.99 (2.68, 6.12)	84.5 (84.2, 84.9)	3.35 (1.07, 11.03)	87.3 (77.6, 93.2)	– –
	50-59y	49.9 (49.3, 50.5)	4.06 (2.67, 6.04)	87.2 (86.9, 87.5)	3.53 (1.12, 11.11)	92.8 (84.2, 96.8)	– –
	60-69y	61.8 (61.2, 62.4)	4.23 (2.76, 6.28)	91.6 (91.4, 91.8)	3.97 (1.35, 11.63)	89.7 (84.0, 93.5)	– –
	70-79y	73.4 (72.9, 73.8)	4.5 (3.02, 6.66)	93.1 (92.8, 93.3)	4.36 (1.41, 13.42)	93.1 (88.4, 95.9)	– –
	80-89y	81.6 (81.2, 82.0)	4.52 (2.97, 6.62)	92.7 (90.7, 94.4)	4.41 (1.40, 12.67)	96.3 (92.6, 98.3)	– –
	≥90y	85.0 (83.6, 86.4)	4.69 (3.11, 7.12)	95.1 (90.0, 97.6)	4.40 (1.27, 13.52)	94.6 (86.7, 97.9)	– –
Urgent care (or higher)							
	0-17y	47.7 (46.1, 49.2)	3.12 (2.34, 4.12)	74.5 (74.0, 75.0)	3.25 (1.47, 7.16)	75.7 (74.0, 77.3)	– –
	18-49y	35.8 (35.1, 36.4)	3.74 (2.51, 5.59)	75.1 (74.7, 75.5)	3.37 (1.15, 10.23)	77.5 (66.3, 85.2)	– –
	50-59y	43.5 (42.5, 44.6)	3.89 (2.62, 5.96)	78.2 (77.7, 78.6)	3.56 (1.19, 11.49)	89.2 (81.1, 94.3)	– –
	60-69y	52.9 (51.9, 54.0)	4.12 (2.77, 6.01)	82.3 (81.9, 82.7)	4.00 (1.27, 12.84)	84.1 (77.9, 89.0)	– –
	70-79y	64.9 (63.8, 66.0)	4.51 (2.91, 6.53)	84.9 (84.5, 85.3)	4.38 (1.26, 12.74)	90.2 (85.3, 93.7)	– –
	80-89y	74.9 (73.7, 76.3)	4.62 (3.04, 6.88)	85.1 (82.2, 87.5)	4.46 (1.38, 13.86)	93.7 (89.5, 96.4)	– –
	≥90y	79.6 (77.0, 81.8)	4.66 (3.06, 6.96)	92.9 (87.6, 96.1)	4.69 (1.46, 13.47)	91.9 (83.9, 96.4)	– –
Emergency department (*or higher*)							
	0-17y	25.7 (24.4, 27.1)	2.94 (2.20, 3.93)	34.9 (34.4, 35.4)	3.37 (1.60, 7.45)	64.0 (62.2, 65.9)	– –
	18-49y	15.8 (15.3, 16.2)	4.10 (2.70, 6.26)	32.7 (32.1, 33.3)	3.71 (1.35, 9.90)	73.2 (61.4, 81.6)	– –
	50-59y	20.4 (19.5, 21.3)	4.41 (2.98, 6.65)	35.4 (34.7, 36.0)	4.14 (1.43, 11.39)	83.1 (73.6, 89.6)	– –
	60-69y	28.3 (27.4, 29.4)	4.74 (3.22, 7.21)	43.5 (42.8, 44.2)	4.62 (1.59, 13.51)	82.8 (75.9, 88.0)	– –
	70-79y	44.6 (43.5, 45.8)	4.93 (3.29, 7.50)	57.3 (56.5, 57.9)	5.02 (1.82, 14.63)	86.2 (80.5, 90.6)	– –
	80-89y	62.6 (61.0, 63.9)	4.82 (3.13, 7.49)	68.9 (68.2, 69.4)	4.83 (1.71, 14.30)	92.7 (88.2, 95.5)	– –
	≥90y	73.6 (71.0, 76.0)	4.78 (3.28, 6.99)	85.2 (78.2, 90.0)	4.92 (1.86, 14.40)	89.2 (80.3, 94.6)	– –
Inpatient admission (*or higher*)							
	0-17y	1.7 (1.4, 2.2)	6.34 (4.11, 10.47)	1.5 (1.2, 1.8)	6.59 (3.28, 13.17)	14.7 (12.9, 16.6)	– –
	18-49y	1.8 (1.7, 2.0)	6.46 (3.49, 12.48)	2.6 (2.5, 2.7)	5.49 (2.00, 14.65)	38.0 (27.8, 49.2)	– –
	50-59y	3.6 (3.2, 4.0)	7.23 (3.69, 13.84)	4.1 (3.4, 4.9)	6.69 (2.50, 18.24)	56.6 (46.8, 66.2)	– –
	60-69y	7.9 (7.3, 8.6)	7.3 (3.88, 13.9)	9.8 (9.4, 10.1)	7.37 (2.88, 17.67)	51.7 (44.3, 59.5)	– –
	70-79y	16.3 (15.4, 17.1)	7.15 (3.71, 13.56)	19.2 (18.7, 19.7)	6.60 (2.63, 17.37)	55.2 (48.8, 61.7)	– –
	80-89y	28.9 (27.5, 30.2)	6.48 (3.53, 12.63)	31.1 (29.8, 32.4)	6.45 (2.67, 17.89)	64.4 (58.2, 69.9)	– –
	≥90y	42.1 (39.5, 45.0)	6.73 (3.53, 12.29)	45.7 (37.7, 53.5)	8.06 (2.93, 21.99)	62.2 (51.3, 72.0)	– –
Mechanical ventilation (*or higher*)							
	0-17y	0.0 (0, 0.2)	– –	0.1 (0.0, 0.2)	– –	0.5 (0.2, 1.1)	– –
	18-49y	0.2 (0.1, 0.3)	– –	0.3 (0.2, 0.5)	– –	4.2 (1.4, 12.1)	– –
	50-59y	0.4 (0.3, 0.6)	– –	0.4 (0.2, 0.7)	– –	7.2 (3.3, 15.2)	– –
	60-69y	1.4 (1.4, 1.5)	– –	1.5 (1.0, 2.0)	– –	6.9 (3.8, 11.8)	– –
	70-79y	3.3 (3.2, 3.4)	– –	2.6 (2.0, 3.4)	– –	8.0 (4.8, 13.5)	– –
	80-89y	7.0 (6.8, 7.2)	– –	5.9 (4.7, 7.8)	– –	8.4 (5.0, 13.2)	– –
	≥90y	13.7 (13.2, 14.2)	– –	14.8 (9.9, 21.0)	– –	20.3 (12.8, 30.4)	– –
Death							
	0-17y	– –	– –	0.0 (0.0, 0.2)	– –	– –	– –
	18-49y	0.1 (0, 0.1)	21.28 (11.08, 39.81)	0.1 (0.1, 0.2)	– –	– –	– –
	50-59y	0.3 (0.2, 0.5)	26.21 (14.08, 47.48)	0.1 (0.0, 0.4)	– –	2.4 (0.6, 8.9)	– –
	60-69y	0.9 (0.9, 1.0)	25.27 (13.37, 49.13)	0.7 (0.4, 1.2)	– –	1.4 (0.3, 5.1)	– –

	70-79y	2.5 (2.4, 2.6)	24.98 (12.57, 48.67)	1.8 (1.3, 2.4)	– –	4.0 (1.9, 7.8)	– –
	80-89y	6.4 (6.2, 6.6)	23.15 (12.75, 45.64)	4.6 (3.5, 6.3)	– –	5.0 (3.0, 10.3)	– –
	≥90y	13.3 (12.9, 13.7)	33.80 (21.09, 53.00)	14.8 (10.2, 20.8)	– –	16.2 (9.5, 26.6)	– –

We report estimates from best-fitting distributions, based on models yielding the minimum AIC score. Missing time-to-event estimates indicate either a lack of model convergence or a confidence interval wider than the follow-up period.
