# Supplementary figures and images for: Clinical progression parameters associated with SARS-CoV-2, influenza, and respiratory syncytial virus infections in a large US integrated healthcare population
Source: PLoS Comput Biol. 2025 Nov 19;21(11):e1013723. doi: 10.1371/journal.pcbi.1013723 (PMC12643285; doi:10.1371/journal.pcbi.1013723)

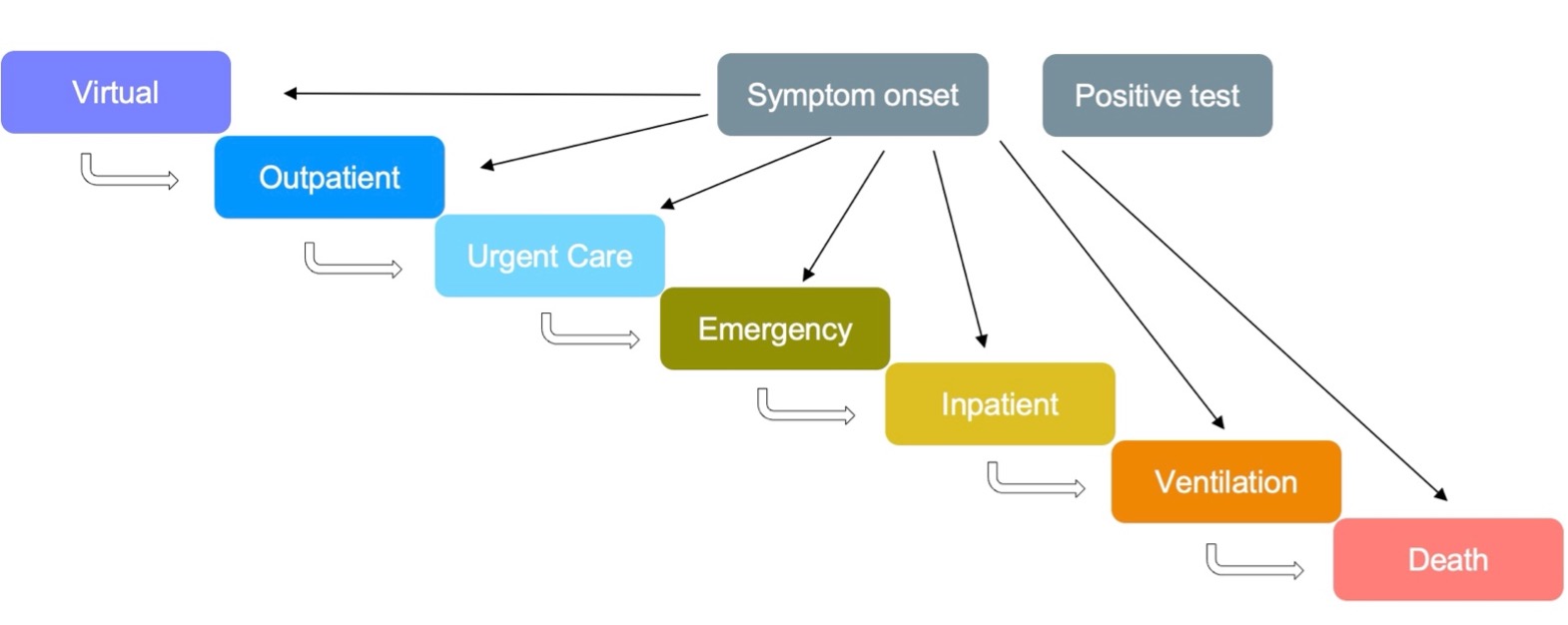

Supplement: S1 File — (ZIP) [file pcbi.1013723.s001.zip › S1 File/S1_Fig.jpg]

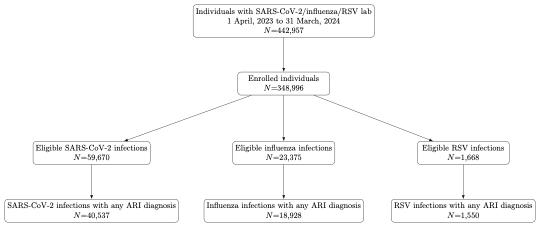

Supplement: S1 File — (ZIP) [file pcbi.1013723.s001.zip › S1 File/S2_Fig.png]
